# Assembly of protein complexes restricts diffusion of Wnt3a proteins

**DOI:** 10.1038/s42003-018-0172-x

**Published:** 2018-10-10

**Authors:** Ritsuko Takada, Yusuke Mii, Elena Krayukhina, Yuusuke Maruyama, Kazuhiro Mio, Yoshikazu Sasaki, Takao Shinkawa, Chan-Gi Pack, Yasushi Sako, Chikara Sato, Susumu Uchiyama, Shinji Takada

**Affiliations:** 1 Exploratory Research Center on Life and Living Systems (ExCELLS), National Institutes of Natural Sciences, 5-1 Higashiyama, Myodaiji, Okazaki, Aichi 444-8787 Japan; 2 National Institute for Basic Biology, National Institutes of Natural Sciences, 5-1 Higashiyama, Myodaiji, Okazaki, Aichi 444-8787 Japan; 3 The Graduate University for Advanced Studies (SOKENDAI), 5-1 Higashiyama, Myodaiji, Okazaki, Aichi 444-8787 Japan; 4 U-Medico Inc., 2-1 Yamadaoka, Suita, Osaka 565-0871 Japan; 5 Graduate School of Engineering, Osaka University, 2-1 Yamadaoka, Suita, Osaka 565-0871 Japan; 6 Biomedical Research Institute, National Institute of Advanced Industrial Science and Technology (AIST), 1-1-1 Higashi, Tsukuba, Ibaraki 305-8566 Japan; 7 Molecular Profiling Research Center for Drug Discovery and OPERANDO Open Innovation Laboratory, National Institute of Advanced Industrial Science and Technology (AIST), 2-3-26 Aomi, Koto-ku, Tokyo 135-0064 Japan; 8 Field Solution Division, JEOL Ltd., 1156 Nakagami, Akishima, Tokyo 196-0022 Japan; 9 BioNet Laboratory Inc., 2-3-28 Nishiki-chou, Tachikawa, Tokyo 190-0022 Japan; 10 Cluster for Pioneering Research, Cellular Informatics Laboratory, RIKEN, 2-1 Hirosawa, Wako, Saitama, 351-0198 Japan; 11 Asan Institute for Life Sciences, Asan Medical Center & University of Ulsan College of Medicine, 88 Olympic-ro 43-gil, Songpa-Gu, Seoul 05505 Republic of Korea

## Abstract

Members of the Wnt protein family play roles in many aspects of embryogenesis and homeostasis. Despite their biological significance, characteristics of Wnt proteins still remain unclear, mainly due to their insolubility after the removal of serum. Here we examine Wnt proteins in serum-containing media by using analytical ultracentrifugation with a fluorescence detection system. This analysis reveals that Wnt3a assembles into high-molecular-weight complexes that become dissociable by interaction with the extracellular domain of the Frizzled8 receptor or secreted Wnt-binding protein sFRP2. Cross-linking and single-particle analyses of Wnt3a fractionated by gel filtration chromatography show the homo-trimer to be the smallest form of the assembled Wnt3a complexes. Fluorescence correlation spectroscopy and immunohistochemistry reveal that the assembly of Wnt3a complexes restricted their diffusion and signaling range in *Xenopus laevis* embryos. Thus, we propose that the Wnt diffusion range can be controlled by a balance between the assembly of Wnt complexes and their dissociation.

## Abstract (web summary)

Ritsuko Takada et al. show that Wnt3a assembles into high molecular weight complexes that restrict the diffusion of Wnt within *Xenopus* embryos. These results suggest that Wnt diffusion in cells is controlled by a balance between higher order complex assembly and dissociation by Wnt-binding proteins.

## Introduction

Wnt is a secreted signal protein, post-translationally modified with palmitoleic acid^1–3^. While it has been shown that Wnt proteins might act at a distance from their source cells, there are also data suggesting that they might act locally as well^4–6^. Thus, if we want to discuss the function of Wnt signals in a particular tissue or cellular context of interest, the range of these signals should require careful consideration. However, the molecular and cellular basis of Wnt short- and long-range actions remains to be elucidated.

Various models have been proposed for Wnt transport between producing and receiving cells. For instance, transcytosis has long been considered as a mechanism for intercellular Wnt trafficking. In addition, specific Wnt-binding proteins, including Swim in *Drosophila*^7^ and secreted Frizzled-related proteins (sFRPs) in vertebrates^8–11^, were also reported to be involved in extracellular Wnt trafficking. Recent studies by imaging analysis also indicated that Wnt transport can be mediated by filopodium-like protrusions^12–15^. On the other hand, it has been suggested that various extracellular deliverers associating with Wnt are involved in Wnt trafficking, e.g., extracellular membranous deliverers, including lipoprotein particles^16,17^ and exosomes^18–23^. Given that these various machineries appear to modulate the Wnt signaling range differently, it is important to reveal how Wnt behaves with respect to each of these machineries after its production and during intercellular transport.

Biochemical characterization is a powerful approach for understanding the molecular basis of protein behavior. Until now, biochemical analyses of Wnt have been mostly carried out by using cell lines in which it is stably produced and efficiently secreted^24^. Wnts secreted from such cell lines can be purified as monomers. However, as the addition of detergent is essential at the beginning and during purification, it is unclear whether monomeric Wnt actually exists in the native condition^25^. In contrast, a gel filtration chromatography study showed that Wnt forms high-molecular-weight (HMW) complexes^25^. However, it is uncertain whether these forms of Wnt naturally exist in culture media. Recently, it was shown that, under conditions where detergent was not added, a considerable amount of Wnt proteins form stable hetero-dimers with afamin, an albumin-like serum component^26^. However, since afamin is not ubiquitously expressed in tissues, it is unlikely that most Wnts actually associate with afamin in the extracellular milieu in vivo. Thus, to be able to apply the results of these biochemical analyses to the understanding of Wnt behavior in vivo, it is desirable to examine Wnt proteins under conditions as close as possible to their native cellular environment. Particularly, sample manipulation, such as addition of detergent and application of chromatography, should be avoided; and effects of serum components should be carefully considered.

In this study, we first examined Wnt proteins in media conditioned by Wnt3a-expressing cells without performing any invasive procedure, including addition of detergent, artificial concentration or chromatographic separation. Analytical ultracentrifugation coupled with a fluorescence detection system (AUC-FDS) allowed us to directly monitor the size of GFP-tagged Wnt3a in the conditioned medium. By this direct analysis, we detected both an afamin-associated form and a HMW form, but not the monomeric form of GFP-tagged Wnt3a, in the conditioned medium. Since the HMW form, but not the afamin complex, was detected independently of serum proteins, we further examined HMW complexes of Wnt3a by utilizing several different techniques, both in vitro and in vivo. These studies reveal a structural basis for Wnt assembly and the significance of this assembly in terms of the regulation of the Wnt signaling range.

## Results

### Different Wnt3a complexes are secreted from cultured cells

To directly monitor Wnt proteins in the conditioned medium, we established a stable L cell line expressing Wnt3a tagged with monomeric EGFP (GFP-Wnt3a), as well as a control line expressing EGFP fused with a signal sequence (secreted GFP). The GFP-Wnt3a retained its signaling activity, although it was partially diminished compared with that of the untagged control (Supplementary Fig. 1). First, we directly examined the average size of GFP-Wnt3a in the conditioned medium by measuring the diffusion coefficient of fluorescent particles by use of Fluorescence Correlation Spectroscopy (FCS). The average size of GFP-Wnt3a, estimated by comparison with the diffusion coefficient of secreted GFP, was apparently larger than that predicted for the monomer, although its molecular mass could not be precisely determined due to the limitation of this technique (Supplementary Fig. 2a, b). In addition, whereas the resulting size did not change considerably upon further incubation for up to 8 days in the conditioned medium containing 8% bovine serum, it gradually increased in the absence of serum (Supplementary Fig. 2c). Thus, Wnt3a proteins, most of which did not appear to exist as a monomer in the conditioned medium, were further assembled into larger complexes during incubation without serum.

**Fig. 1:**
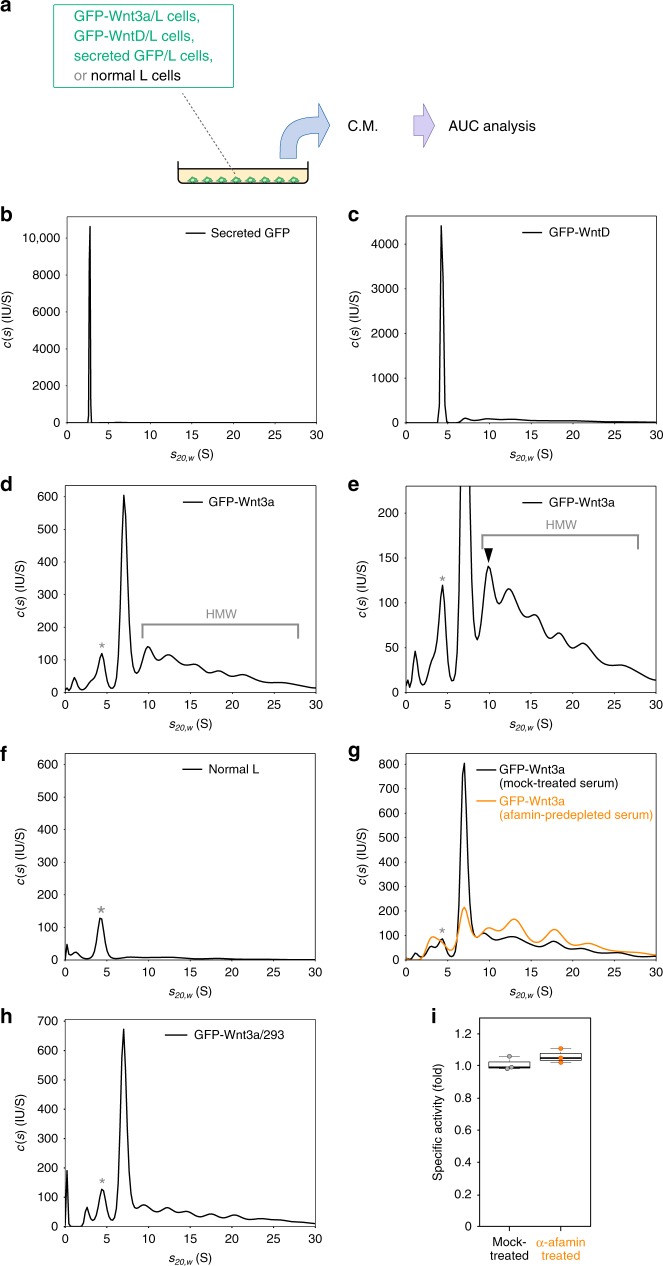
Analytical ultracentrifugation analyses of GFP-tagged Wnt3a proteins secreted from cultured cells. **a** Schematic representation of the analysis using AUC. **b**–**f** AUC-FDS analysis of secreted GFP (*n* = 3; (**b**)), GFP-tagged Drosophila WntD (GFP-WntD; *n* = 7; (**c**)), and GFP-tagged mouse Wnt3a (GFP-Wnt3a; *n* = 20; (**d**, **e**)), secreted from stably transformed mouse L cells. As a negative control, AUC-FDS analysis of conditioned medium (C.M.) from neomycin-resistant L cells is also indicated (*n* = 4; (**f**)); **e** is an enlarged version of **d**. HMW indicates high-molecular-weight complex. The closed arrowhead in **e** indicates the HMW complex with the smallest molecular mass (~9.6 S corresponding to ~200 kDa). **g** AUC-FDS analysis of GFP-Wnt3a in culture media with mock-treated (black; n = 3) or afamin pre-depleted (orange; n = 3) serum. The relative area size of the Wnt3a/afamin complex peak, which was normalized by the whole area size obtained in each measurement, was significantly decreased by afamin pre-depletion (relative area of ~ 7.0 peaks was decreased from 35.1 ± 6.6% to 14.1 ± 1.5% by the pre-depletion (mean ± s.d., *p* *<* 0.05); whereas that of the HMW peaks was increased from 51.1 ± 4.8% to 69.6 ± 1.2% (mean ± s.d., *p* *<* 0.05, *n* = 3, paired *t-*test). **h** AUC-FDS analysis of GFP-Wnt3a secreted from HEK293 cells (*n* = 4). **i** Wnt signaling activity of GFP-Wnt3a in culture media with mock-treated (black) or afamin pre-depleted (orange) serum. Wnt activity monitored by use of the SuperTOPFlash assay was normalized by the fluorescence intensity of GFP. Peaks marked with asterisks in **d**–**h** appeared to be derived from fluorescent molecules bound to serum albumin, such as bilirubin (Supplementary Fig. 3f)

To further examine Wnt proteins by non-invasive procedure, we next used AUC-FDS, which allowed us to monitor the sedimentation of GFP-tagged proteins even in the presence of serum proteins (Fig. 1). In addition to GFP-Wnt3a and secreted GFP, we also examined EGFP-tagged *Drosophila* WntD (GFP-WntD), which lacks a palmitoleoylation site conserved among most members of the Wnt family proteins^27–29^, in this experiment. In the culture medium, the *c*(*s*) distribution of secreted GFP showed one major peak with a sedimentation coefficient of ~2.8 S (Fig. 1b). This peak was assigned to the monomeric form of secreted GFP because its apparent molecular mass of ~30 kDa well corresponded to the molecular mass of the monomeric GFP calculated from its amino-acid sequence (28 kDa). Likewise, GFP-WntD showed 1 major peak at ~4.1 S (Fig. 1c), which was attributed to the monomeric GFP-WntD (calculated molecular mass: 61 kDa). On the other hand, GFP-Wnt3a exhibited a major peak at ~7.0 S with the apparent molecular mass of ~150 kDa as well as widely-distributed HMW complexes (Fig. 1d). In these HMW complexes, a peak with the smallest molecular mass (closed arrowhead) was detected around ~9.6 S with an apparent molecular mass of ~200 kDa (Fig. 1d, e). Whereas the position of this peak was only slightly affected by dilution of the medium, that of the other peaks in the HMW complexes were more evidently shifted (Supplementary Fig. 3j). Thus, we could not estimate the molecular size from the position of the other peaks in the HMW complexes. We also confirmed secretion of similar peaks when we expressed GFP-Wnt3a in HEK 293 cells (Fig. 1h).

Importantly, although a small peak was detected at ~4.2 S (marked by an asterisk in Fig. 1d), which was close to the size of the monomeric form of GFP-Wnt3a (molecular mass of the glycosylated and palmitoleoylated form: 70 kDa), a similar peak was also detected even in the culture medium of normal L cells (Fig. 1f). Furthermore, this 4.2 S peak appeared in a manner depending on the addition of albumin to serum-free medium (Supplementary Fig. 3f). Because the apparent molecular mass corresponding to this 4.2 S peak corresponded closely to the calculated molecular mass of bovine albumin (66 kDa), this fluorescence was likely to be derived from fluorescent molecules that binds to albumin in the serum, such as bilirubin. To further confirm these results, we analyzed GFP-Wnt3a proteins prepared from medium conditioned by the Wnt-producing cells for 2 days after removal of serum (Supplementary Fig. 3g, h). Although the overall amount of secreted GFP-Wnt3a proteins was decreased under this condition, probably due to shortage of serum-derived nutrients, several peaks in the HMW complexes were still detectable. In contrast, the 4.2 S peak was not apparent under this condition (Supplementary Fig. 3g). Taken together, we concluded that no significant amount of the monomeric form of GFP-Wnt3a was detected in the conditioned medium.

It was already shown that a large portion of Wnt3a secreted into the culture medium forms a 1:1 complex with afamin, a serum protein^26^. The calculated weight of the GFP-Wnt3a/afamin complex was 140 kDa, suggesting that the 7.0 S peak with an apparent molecular mass of ~150 kDa could correspond to this complex. However, due to concerns that the accuracy of the molecular mass estimate can be compromised in the case of non-single peak distributions, we further tested our assumption by depletion or inactivation of afamin in the serum. The area under the 7.0 S peak was considerably diminished when afamin was immuno-depleted from the serum prior to the cultivation with GFP-Wnt3a expressing cells (Fig. 1g). Furthermore, the area under the 7.0 S peak of GFP-Wnt3a was specifically reduced by preincubation of serum at 56 °C prior to cultivation with GFP-Wnt3a-producing cells (Supplementary Fig. 3d), but this reduction was recovered by addition of purified human afamin to the culture medium (Supplementary Fig. 3e). In contrast, afamin-depletion did not have any obvious effect on the monomer peak of GFP-WntD, as predicted (Supplementary Fig. 3a–c). Thus, GFP-Wnt3a, but not GFP-WntD, secreted into the culture medium formed a complex with afamin.

In contrast, the amount of the HMW complexes of GFP-Wnt3a was not apparently decreased, but slightly increased, by afamin-depletion (Fig. 1g) or preincubation of the serum at 56 °C (Supplementary Fig. 3d). Analysis of GFP-Wnt3a prepared under serum-free culture condition showed that a significant amount of the HMW complexes, including that of the smallest HMW peak, was still detectable under this condition (Supplementary Fig. 3g,h), in addition to a ~3 S peak, which was likely due to degradation of GFP-Wnt3a proteins (Supplementary Fig. 3i). In contrast, the 7.0 S peak corresponding to the Wnt/afamin complex was not apparent as predicted (Supplementary Fig. 3g, h). Although several distinct peaks were evident in serum-free medium, the number of peaks of the HMW complexes was reduced (Supplementary Fig. 3g; compared with Fig. 1d or Supplementary Fig. 3d). Thus, some of the HMW peaks, including the smallest HMW peak, were generated in a serum-independent manner; whereas some of the other peaks might have been formed by association of Wnt3a proteins with as yet unidentified serum components. Of note, the HMW complexes appeared to retain their activity because the signaling activity per GFP-Wnt3a molecule was not significantly changed by depletion of afamin (Fig. 1i).

### Dissociation of Wnt3a complexes by Frizzled8 or sFRP2

**Fig. 2:**
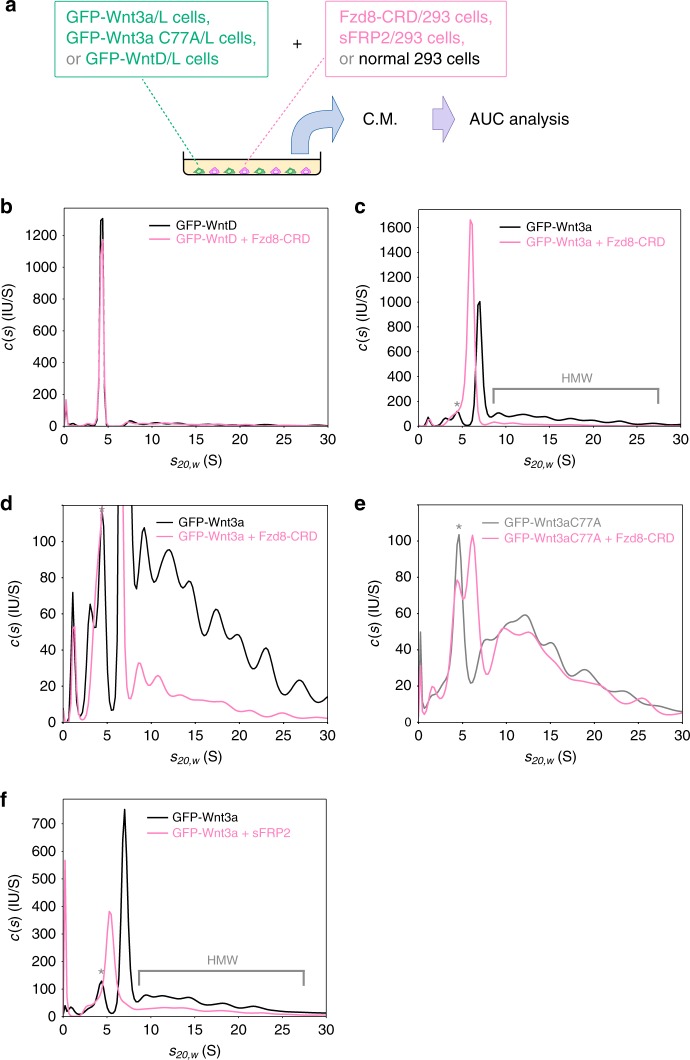
Analytical ultracentrifugation analyses of GFP-tagged Wnt proteins with Fzd8 CRD or sFRP2. **a** Schematic representation of the analysis. **b**, **c** AUC-FDS analysis of conditioned medium of L cells secreting GFP-WntD (*n* = 7; (**b**)) and GFP-Wnt3a (*n* = 8; (**c**)) co-cultured with HEK293 cells secreting the CRD domain of mouse Fzd8 (pink line) or with control HEK293 cells (black line). For comparison with (**e**) below, an enlargement of (**c**) is also shown as (**d**). **e** AUC-FDS analysis of conditioned medium of L cells secreting GFP-Wnt3a carrying the C77A mutation cultured with HEK293 cells secreting the CRD domain of mouse Fzd8 (pink line; *n* = 3) or control HEK293 (gray line; *n* = 3). A peak with molecular mass corresponding to the Wnt/Fzd8-CRD complex was detected, but its area was smaller comapred with the case of wild-type Wnt3a. **f** AUC-FDS analysis of conditioned medium of L cells secreting GFP-Wnt3a cultured with HEK293 cells secreting mouse sFRP2 (pink line; *n* = 8) or control HEK293 cells (black line; *n* = 8). HMW indicates high-molecular-weight complex. Peaks marked with asterisks in **c**–**f** appeared to be derived from fluorescent molecules bound to serum albumin, such as bilirubin

We next examined whether the Wnt/afamin and the HMW complexes could bind to their receptor, Frizzled (Fzd). For this purpose, GFP-Wnt3a-expressing L cells were co-cultured with HEK293 cells secreting a truncated form of mouse Fzd8 containing the CRD domain (Fzd8-CRD), which binds to Wnt ligands, but lacking the trans-membrane and cytoplasmic domains (Fig. 2a). The *c*(*s*) distribution of GFP-WntD was notchanged by co-culture with Fzd8-CRD-expressing cells (Fig. 2b). In contrast, in the *c*(*s*) distribution obtained from the  co-culture of GFP-Wnt3a cells with Fzd8-CRD-expressing cells, a major peak at ~6.0 S emerged concomitant with a decrease in the area under the Wnt3a/afamin and HMW complexes peaks (Fig. 2c, d). Thus, Wnt3a in both the Wnt3a/afamin complex and HMW complexes could interact with the Fzd8-CRD. We conclude that both forms of Wnt3a could specifically bind with the Fzd8 receptor after secretion into the medium.

Since Wnt3a can associate with sFRPs (soluble Frizzled-related proteins), we also examined the binding of the 2 distinct forms of Wnt3a with mouse sFRP2. Similar to the case of Fzd8-CRD, a decrease in the area under Wnt3a/afamin and HMW complex peaks was accompanied by the appearance of a major peak at ~5.4 S; although HMW complex peaks were decreased to a lesser extent (Fig. 2f). Quantification of the relative size of the area of HMW complex peaks, which was normalized with the whole area obtained in each measurement, indicated a significant decrease in HMW complex peaks (relative area of HMG peaks was decreased from 46.4 ± 0.8% to 33.6 ± 2.9% by the addition of sFRP2; mean ± s.d., *n* = 8; *p* *<* 0.001, paired *t-*test). Thus, sFRP2 reduced the Wnt3a/afamin complex and the HMW complex, although there was a difference in the degree of influence. Even though the calculated molecular mass of sFRP2 (33 kDa) was slightly larger than that of Fzd8-CRD (27 kDa), the *s*_*20,w*_-value detected for the co-culture with sFRP2-expressing cells (~5.4 S) was smaller than that with Fzd8-CRD-expressing ones (~6.0 S). We assume that this reversion was probably due to a difference in molecular shape or stoichiometry of components between the 2 complexes.

### HMW complexes are different from artificial Wnt oligomers

Oligomer formation of Wnt was previously reported in the case where the N-terminus sequence of mouse Wnt3a is cleaved by a protease called Tiki or where a conserved cysteine residue, C77 of mouse Wnt3a, is mutated^30^. However, these oligomers are not active after secretion into the medium^25,30^. In an attempt to compare the previous studies with our present one, we subjected a mouse Wnt3a mutant, in which C77 was substituted to alanine, to AUC-FDS. In the *c*(*s*) distribution of this mutant, the formation of the Wnt3a/afamin complex was markedly decreased (gray line in Fig. 2e), whereas a wide distribution of peaks with larger sedimentation coefficients, assumingly corresponding to homo-oligomers, was observed. However, in contrast to the HMW complexes of wild-type Wnt3a, these C77A Wnt3a oligomers less efficiently bound to Fzd8-CRD and were less prone to undergo dissociation (pink line in Fig. 2e). Thus, the HMW complexes composed of wild-type Wnt3a were distinct from the previously reported oligomers in terms of dissociability.

### Wnt trimer is smallest form of HMW complexes

**Fig. 3:**
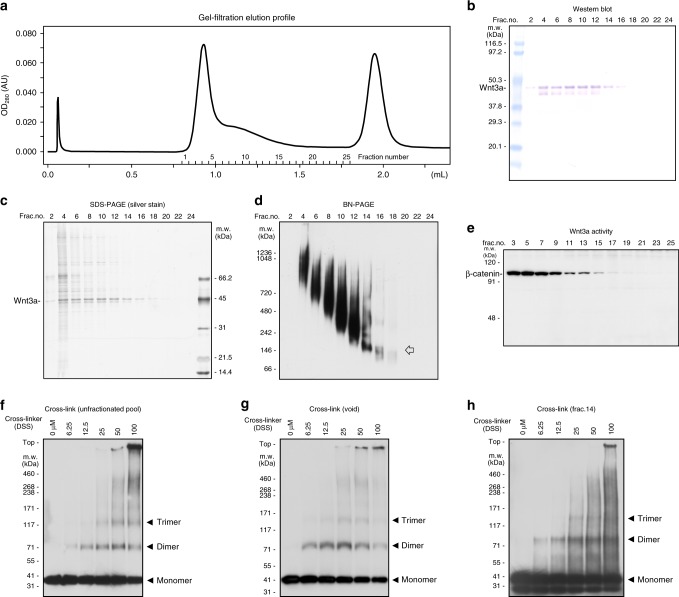
Characterization of Wnt3a fractionated by gel filtration. **a** Elution profile of gel filtration column chromatography. Affinity-purified FLAG-Wnt3a proteins, prepared from serum-free medium conditioned with FLAG-Wnt3a-secreting L cells, were subjected to gel filtration column chromatography (Superdex200 PC3.2/30, GE Healthcare). **b** Analysis of fractionated proteins by western blotting with anti-FLAG antibody. Wnt3a proteins are recovered in widely spread fractions, including a fraction corresponded to the void volume (fraction #4). **c** Analysis of fractionated proteins by silver staining. Protein whose size corresponded to FLAG-Wnt3a is the major component in fractions from fraction #2 to #16. **d** Analysis of fractionated proteins by western blotting using Blue Native PAGE (Invitrogen), which can separate high-molecular-weight proteins while maintaining their native conformation. This analysis shows that the size of Wnt-3a protein complex is mostly distributed between ~150 kDa and 1000 kDa. Of note, a discrete band corresponding to 150 kDa is detected in fraction 14. An arrow indicates a discrete band whose molecular weight corresponded to the size of a Wnt trimer. **e** Wnt signaling activity assessed by application of each fraction to mouse L cells. Since the β-catenin protein level is quite low in the absence of Wnt signaling in L cells, we directly monitored Wnt signaling activity by measuring the β-catenin protein level in these L cells. All of the fractions in which FLAG-Wnt3a was detectable increased the β-catenin level, indicating that Wnt3a protein still possessed its signaling activity. **f**–**h** Cross-linking analysis with proteins in unfractionated pool (**f**), in void volume (**g**), and in fraction 14 (**h**). Full blot images of **b**–**h** are shown in Supplementary Fig. 9

Since some of the HMW complexes natively existed in the conditioned medium even after the removal of the serum, we further examined the HMW complexes by using column chromatography. Although we anticipated that the artificial concentration procedure for sample preparation or chromatographic separation would enhance Wnt3a assembly, we decided nonetheless to perform this experiment because we expected that column chromatography could allow us to characterize some particular population of the HMW complexes, especially the smallest HMW complex (closed arrowhead in Fig. 1e), separately from the other HMW complexes. To this end, we used an L cell line that stably produced FLAG-tagged Wnt3a (FLAG-Wnt3a), which have a reduced steric effect compared with the GFP-tagged Wnt3a. To exclude serum components from the analysis, we prepared proteins from the medium conditioned by Wnt-expressing cells for 2 days after serum removal. After affinity purification with anti-FLAG antibody, purified FLAG-Wnt3a proteins were separated by gel filtration without the addition of CHAPS (Fig. 3a). FLAG-Wnt3a proteins were eluted in the void volume and also recovered in wide-spread fractions, indicating that they were heterogeneous in size (Fig. 3b). Western blotting of each fraction by using Blue Native PAGE (Invitrogen), which can separate protein complexes while maintaining their native conformation, indicated that the size of the FLAG-Wnt3a protein complex was gradually distributed between ~150 kDa and 1000 kDa (Fig. 3d). Remarkably, FLAG-Wnt3a proteins distributed in this range still possessed signaling activity, as measured by stabilization of β-catenin proteins (Fig. 3e).

**Fig. 4:**
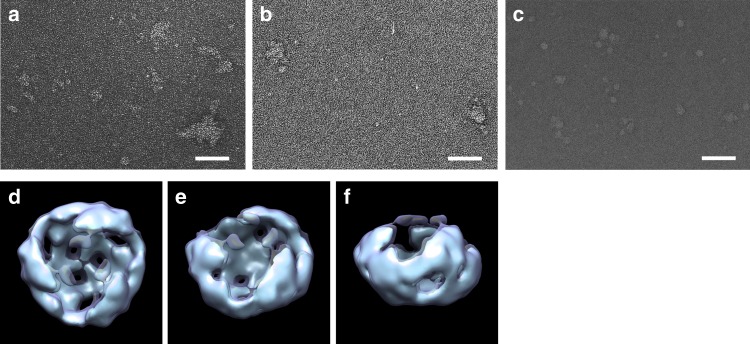
Electron microscopic images of fractionated Wnt3a. **a**–**c** EM images of unfractionated sample (**a**), sample eluted in void volume (**b**), and sample recovered in fraction 14 (**c**). The scale bar in **a**–**c** represents 500 Å. **d**–**f** Image of Wnt3a reconstructed by single-particle analysis. Top view (**d**), oblique view (**e**), and side view (**f**) are shown. The volume enclosed by the isosurface is 100% (cyan) and 150% (dark blue) of the volume estimated from the molecular weight of the Wnt3a trimer

Interestingly, FLAG-Wnt3a proteins recovered in fraction 14, where small forms of Wnt3a were recovered, exhibited a distinct band upon Blue Native PAGE analysis (Fig. 3d). Electron microscopic (EM) images of protein particles in each fraction (Fig. 4a–c) indicated that the size and shape of most of the particles in the Wnt3a pool before the gel filtration and those eluted in the void volume were relatively large and quite heterogeneous (Fig. 4a, b). In contrast, those in fraction 14 looked relatively uniform (Fig. 4c), consistent with the appearance of a distinct band in Blue Native PAGE analysis. Because FLAG-Wnt3a was the dominant protein in fraction 14 (Fig. 3c) and the size of the distinct band detected by Blue Native PAGE corresponded to that of Wn3a trimer, this distinct band appeared to indicate Wnt3a trimer formation (Fig. 3d). To further test this possibility, we next performed cross-linking analysis using fraction 14 (Fig. 3h). By increasing the concentration of the cross-linker, discrete bands up to triple the size of FLAG-Wnt3a were detected, which is consistent with homo-trimer formation of Wnt3a. Of note, these findings are in agreement with the AUC-FDS results, where the apparent molecular mass of the smallest HMW complex (~200 kDa) was similar to that of GFP-Wnt3a homo-trimer (Fig. 1d). All of these findings supported the idea that the homo-trimer was the smallest form in the HMW complexes. In addition, cross-linking analysis of other Wnt3a-containing fractions, including samples eluted in the void volume of the column (Fig. 3g), as well as that with Wnt3a pool before separation by gel filtration (Fig. 3f), also showed results similar to those obtained with fraction 14. These results suggest that the homo-trimer might have been incorporated into larger Wnt3a complexes recovered in other fractions.

Next, we sought to reconstruct the 3D structure of the Wnt trimer from EM images of particles in fraction 14 by performing single-particle analysis (Fig. 4d–f, Supplementary Fig. 4a–c). Prior to this analysis, we reconstructed the 3D structure of one typical particle by electron tomography. The reconstructed image showed a three-fold rotational symmetrical structure (Supplementary Fig. 5a–c), further supporting the formation of the Wnt homo-trimer. Consistently, many raw images of FLAG-Wnt3a showed this three-fold rotational symmetry structure, which we interpreted to be top views. Single-particle analysis using 4,999 images of similar-sized particles in fraction 14, hypothesizing three-fold symmetry, revealed that 3 Wnt3a molecules were arranged on the same circumference of a circle to overlap each other (Fig. 4d–f). Taken together, the results of AUC-FDS analysis, as well as the biochemical and EM-based structural analyses, strongly suggested that the Wnt3a proteins associated with each other to form the homo-trimer, which could further be assembled with like molecules to form larger complexes, at least in the cell culture system.

### Wnt diffusion range is restricted by its assembly in vivo

**Fig. 5:**
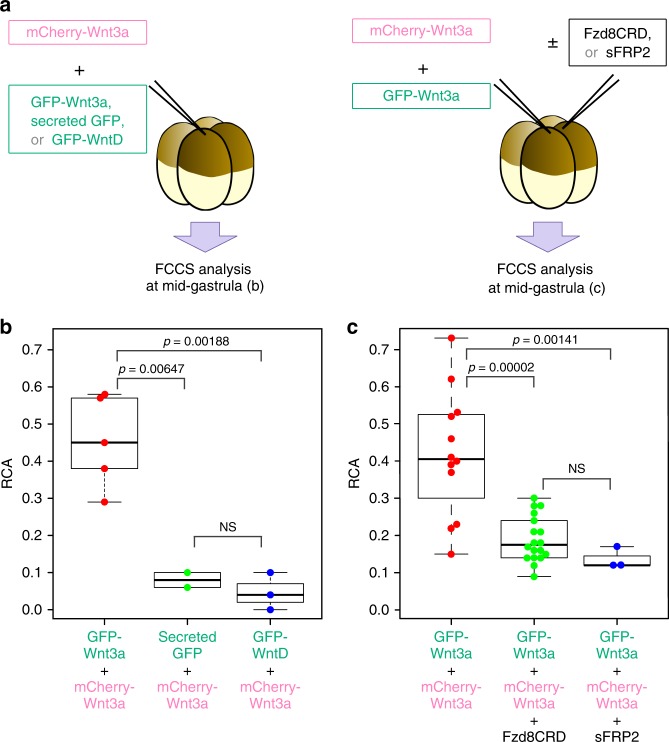
Fluorescence Cross-Correlation Spectroscopy (FCCS) analysis of Wnt3a in the extracellular milieu in *Xenopus* embryos. **a** FCCS analyses of the extracellular milieu within almost one-cell diameter from Wnt3a-expressing cells were carried out in *Xenopus* embryo at the mid-gastrula stage. Through a number of trials of FCCS and FCS (Fig. 6) analyses, we found that the behavior of Wnt proteins was quite different between the inside and outside of cells. Thus, we could ensure the extracellular measurements by examining the dynamics of Wnt proteins. **b** Molecular interaction between mCherry-Wnt3a and GFP-Wnt3a, secreted GFP or GFP-WntD is evaluated with RCA (relative cross-correlation amplitude), which corresponds to the ratio of interacting molecules to all molecules. **c** Effects of overexpression of Fzd8-CRD or sFRP2 on molecular interaction between mCherry-Wnt3a and GFP-Wnt3a. Statistical significance (*p*) was calculated by use of Tukey’s multiple comparisons of means. NS means not significant

The next important question became whether Wnt molecules could actually form complexes with each other in vivo. To address this question, we performed Fluorescence Cross-Correlation Spectroscopy (FCCS), by which we could quantify the interaction of 2 differently labeled-molecules by measuring the correlation of their movements within a confocal volume. We injected mRNAs encoding mCherry-tagged and GFP-tagged Wnt3a into *Xenopus* embryos at the 4-cell stage and then performed FCCS analysis at the gastrula stage (Fig. 5a, Supplementary Fig. 6). Measurement by FCCS was done in the extracellular milieu within almost one-cell diameter from Wnt3a-expressing cells to detect the Wnt3a complex after the secretion. As controls, secreted GFP or GFP-WntD was co-expressed with mCherry-Wnt3a (Fig. 5b). Molecular interaction was evaluated with RCA (relative cross-correlation amplitude), which corresponds to the ratio of interacting molecules to all molecules. The mean RCAs of mCherry-Wnt3a and GFP-Wnt3a was substantially higher than those of mCherry-Wnt3a and the controls (Fig. 5b), indicating that Wnt3a proteins secreted into the extracellular milieu could actually form complexes with each other in vivo. Furthermore, this complex formation was inhibited by expression of Fzd8-CRD or sFRP2 (Fig. 5c), indicating that Wnt3a complex formed in vivo was also dissociable by Fzd8-CRD or sFRP2.

**Fig. 6:**
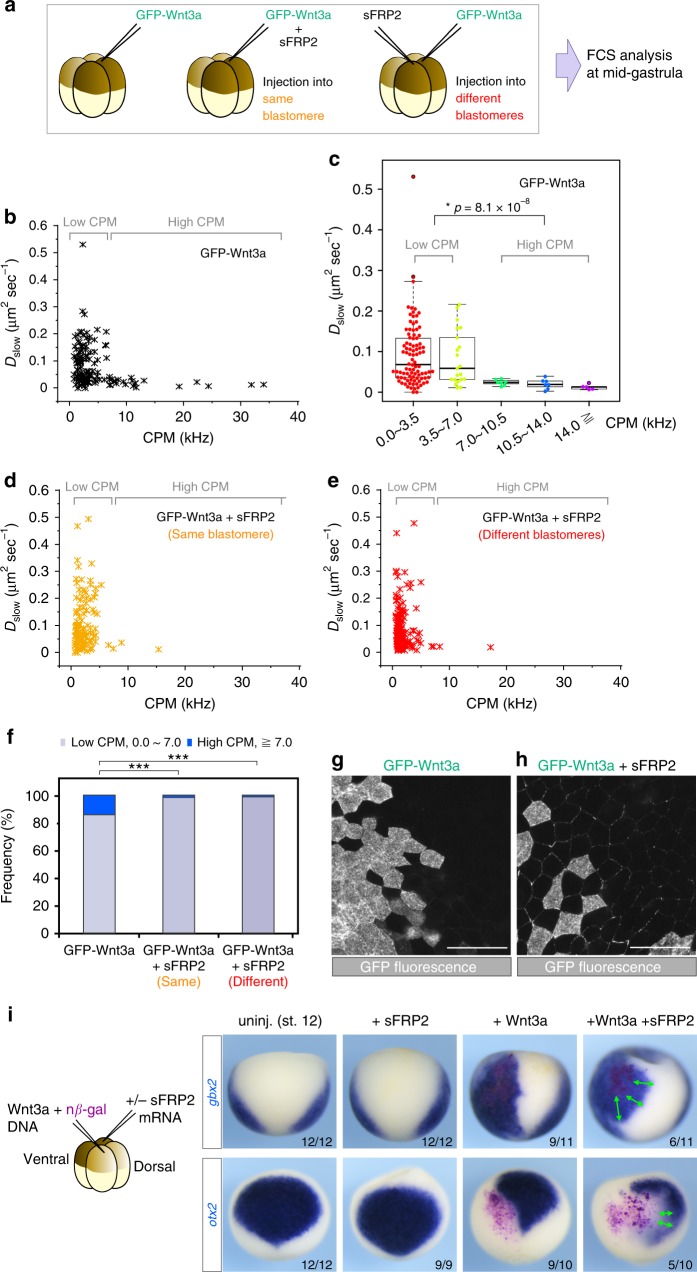
Expansion of Wnt3a distribution range by expression of sFRP2 in *Xenopus* embryos. **a**–**f** Fluorescence Correlation Spectroscopy (FCS) analysis of GFP-Wnt3a. Schematic representation of the FCS analyses is presented (**a**). *GFP-Wnt3a* mRNA was injected into a blastomere (**b**, **c**), and *GFP-Wnt3a* and *sFRP2* mRNAs were injected into the same blastomere (**d**) or into different blastomeres (**e**) in *Xenopus* eggs at the 4-cell stage, and then the extracellular milieu within almost one-cell diameter from Wnt3a-expressing cells was analyzed by FCS at the mid-gastrula stage. CPM and *D*_slow_ measured in each time period (10 seconds) are plotted (**b**, **d**, **e**). Distribution of *D*_slow_ values shown in (**b**) is indicated in increments of 3.5 CPM (**c**). The pattern of distribution of *D*_slow_ was clearly divided at 7.0 CPM. Statistical significance (*p*) was calculated by using the Wilcoxon rank sum test. Summarized representation of the high and low CPM populations is also shown (**f**). Multiple comparisons using Bonferroni correction were performed with Fisher’s exact test (two-sided; ***p *<* 0.001). **g**, **h** Distribution of GFP-Wnt3a in the absence (*n* = 4; (**g**)) or presence (*n* = 4; (**h**)) of sFRP2 expression. *GFP-Wnt3a* and *sFRP2* mRNAs were injected into different blastomeres in *Xenopus* embryos at the 4-cell stage, and the distribution of GFP-Wnt3a was determined by GFP-Fluorescence at stage 10.5. The distribution range of GFP-Wnt3a was expanded in the presence of sFRP2. The scale bars in (**g**), (**h**) represent 100 μm. **i** Expansion of the signaling range of Wnt3a by sFRP2. Wnt3a was expressed by injecting a DNA expression construct (pCS2 + Wnt3a, 8.25 pg/embryo) to avoid affecting the early Wnt signaling involved in the dorsal determination, together with nβ-gal (pCS2 + nβ-gal, 100 pg/embryo). *sFRP2* mRNA (1 pg/embryo) was injected in the blastomere diagonal to the Wnt3a-injected one, as illustrated. Wnt3a source cells were stained in magenta. *In situ hybridization* of Wnt target genes, *gbx2* or *otx2*, was examined in st. 12 *Xenopus* embryos. Double-headed arrows indicate expansion of Wnt3a effect on *gbx2* activation or *otx2* inhibition. Representative images are presented (numbers of embryos with similar staining patterns/total embryos examined are as indicated at the right bottom of panels)

We next examined the mobility of Wnt3a in the extracellular milieu by performing FCS analysis of GFP-Wnt3a in *Xenopus* embryos (Fig. 6a–f, Supplementary Fig. 7). This examination revealed that the dynamic behavior of Wnt3a could be best described by a two-state model (Supplementary Fig. 7e). Correlation analysis yielded 2 components, a fast component with the diffusion coefficient of *D*_fast_ = 12.6 ± 8.8 μm^2^ s^−1^ and a slow component with one of *D*_slow_ = 0.09 ± 0.08 μm^2^ s^−1^ (Supplementary Fig. 7b, e, f). Considering these diffusion coefficients, the fast component, whose fractional ratio was 19 % (Supplementary Fig. 7f), appeared to reflect molecules diffusing freely in the extracellular milieu; and the slow component, whose fractional ratio was 81% (Supplementary Fig. 7f), to reflect molecules moving slowly probably due to interaction with extracellular matrices (ECMs; Supplementary Fig. 7a). However, since an FCS measurement is based on fluorescence fluctuation, molecules that are immobile within the measurement period are not detectable by this method. Therefore, we note that Wnt3a molecules that might have been tightly bound to the extracellular matrix were likely to have been underestimated in this experiment (Supplementary Fig. 7a).

To further analyze the interaction between Wnt3a and the ECM, we focused on the slow component. Interestingly, we noticed that this component could further be divided into 2 distinct populations depending on the count-per-molecule (CPM) value, which correlates with the average number of GFP molecules in complexes. One population could be recognized by relatively low CPM values (<7 CPM). This population showed a variety of diffusion coefficients in embryos expressing GFP-Wnt3a (Fig. 6b, c). Given that diffusion coefficients of the slow component are largely affected by interaction with the ECM, this variety of diffusion coefficients suggests that the period to bind the ECM was stochastically determined in Wnt3a proteins in this population. Although the resolution of this experiment did not allow us to estimate the exact size of Wnt3a particles, particles recovered in this population might have included small-sized homo-complexes and/or Wnt3a proteins associated with some unidentified proteins.

The other population, recognized by relatively high CPM values (≧7 CPM, Fig. 6b, c, indicated by high CPM), consisted of Wnt3a particles containing many Wnt3a molecules. In contrast to the low CPM population, all of Wnt3a particles in the high CPM population showed slow diffusion coefficients (Fig. 6b, c). Thus, these particles were relatively tightly associated with the ECM, although they were still slightly diffusible because their fluctuations were detectable by FCS. Injection of *sFRP2* mRNA in the same blastomere (Fig. 6d) specifically diminished this population (Fig. 6f), suggesting that GFP-Wnt3a formed large assemblies that could be dissociated by their interaction with sFRP2 in vivo, similarly to HMW complexes detected in vitro (Fig. 2f). In addition, sFRP2 increased the number of GFP-Wnt3a particles detectable by FCS (Supplementary Fig. 7f). This increase may have been caused at least partly by dissociation of the large assemblies of GFP-Wnt3a. Furthermore, this dissociation appeared to occur after the secretion of Wnt proteins; because *sFRP2* mRNA injected into different blastomeres gave similar results (Fig. 6e, Supplementary Fig. 7f). Taken together, large assemblies of Wnt proteins, which were dissociable by interaction with sFRP2, had relatively tight associations with the ECM.

Since the large assembled forms of Wnt3a were less mobile in the extracellular milieu, it seems probable that the assembly of Wnt3a restricted its diffusion in embryos. It also seems likely that dissociation of the large assembly by interaction with sFRP2 would generate much more amount of diffusible Wnt3a protein complexes, probably resulting in a longer diffusion distance in the embryo. Thus, we examined the distribution range of Wnt3a in *Xenopus* embryos with or without injection of *sFRP2* mRNA. The spreading of GFP-Wnt3a, as well as the expansion of the signaling range of Wnt3a, was observed by expression with sFRP2 (Fig. 6g–i), as in the case of *Xenopus* Wnt8 and another sFRP member, Frzb^9^. Furthermore, sFRP2 was co-localized with GFP-Wnt3a in the area where the expanded distribution was observed (Supplementary Fig. 8). In contrast, most of the GFP-Wnt3a still remained in the expressing cells in the condition without sFRP2 expression (Fig. 6g). Thus, in *Xenopus* embryos, the diffusion of Wnt3a was tightly restricted without sFRP2, which could dissociate the large Wnt3a assembly.

## Discussion

**Fig. 7:**
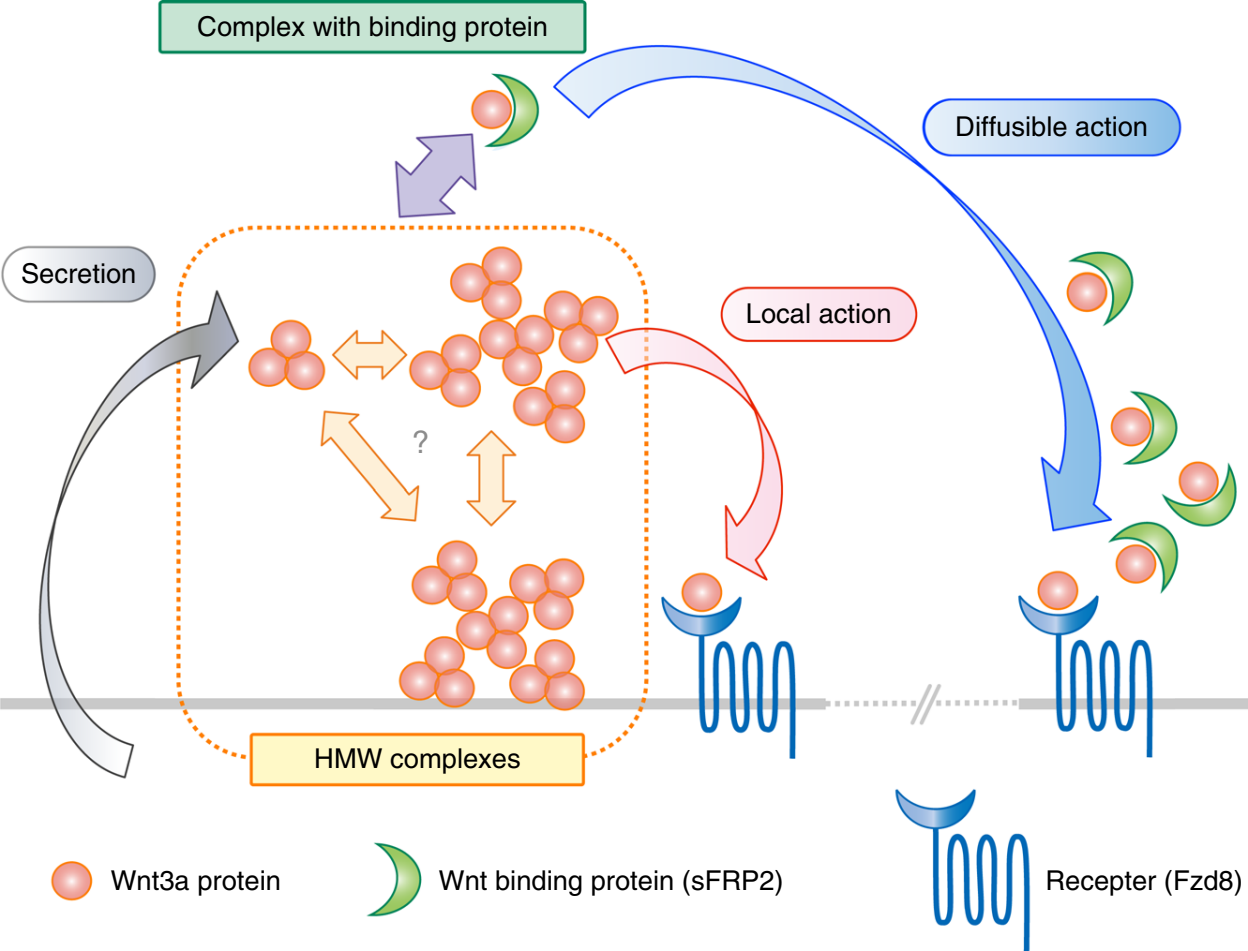
Model: Heterogeneity of Wnt complex formation and diffusion range. Wnt trimer is the smallest unit of the HMW complexes. Both the trimer and trimer-assembled larger complexes appear to exist in the extracellular milieu, although it is uncertain as to when the timing of the trimer formation, as well as that of the assembly to the larger HMW complexes, occurs during the process of Wnt secretion. The large HMW complex is less mobile, probably interacting with the plasma membrane, resulting in restriction of the Wnt diffusion range. Some Wnt molecules in the HMW form can be dissociated by local interaction with Frizzled receptor (Fzd), resulting in short-range signal (local action). In contrast, the larger HMW complexes, probably as well as the trimer itself, can also be dissociated by interaction with soluble Wnt-binding protein, such as sFRP2. By this dissociation, Wnt becomes more mobile; and its diffusion range is thus expanded (diffusible action)

Here we provide evidence that Wnt3a proteins self-associate forming a homo-trimer that can possibly further assemble into higher-order structures. The trimerization and assembly of the Wnt trimers seem to be a plausible way for shielding the lipid adduct of Wnt proteins from the aquatic environment. Of note, the assembled Wnt3a retained signaling activity and could be dissociated by interaction with Fzd8 receptor or with a secreted Wnt-binding protein, sFRP2, in vitro. Consistently, Wnt3a proteins in *Xenopus* embryos could assemble with each other; and Wnt3a assemblies could be dissociated by sFRP2. We also showed that large assemblies of Wnt3a were less mobile and that sFRP2 expanded the diffusion range of Wnt proteins in *Xenopus* embryos. On the basis of these results, we propose a model in which the balance between assembly of the Wnt3a trimers and dissociation of these assemblies, caused by dissociation of the trimers in these assemblies by interaction with Wnt-binding proteins, such as sFRP2, modulates the range of Wnt signaling (Fig. 7).

A number of studies have investigated characteristics of Wnt proteins and reported result that were different from ours. A previous study using gel filtration chromatography reported that active Wnt3a is monomeric^25^. In contrast, in our present study, an appreciable amount of monomeric Wnt3a was detected by neither AUC-FDS nor gel filtration analysis. Rather, our results suggest the Wnt trimer acts as a basic assembly unit. Apparently, there are several differences in experimental design between the previous study and ours. The most critical difference is that the Wnt3a proteins examined in the previous study were prepared in the presence of the detergent CHAPS. We assume that the hydrophobic lipid adduct on monomeric Wnt3a could be stabilized in the previous study probably due to the effect of detergent. On the other hand, as discussed above, we speculate that the lipid adducts could be shielded by the trimerization of Wnt3a in the native condition. Therefore, it is reasonable to consider that most Wnt3a proteins in the conditioned medium did not exist as monomers in the absence of detergent.

Wnt proteins are known to aggregate in solution unless stabilized by a detergent or serum. Since the purified Wnt3a proteins lose their activities when they are incubated in serum-free medium^31^; however, it has long been considered that large complexes of Wnt proteins are inactive^25^. In contrast, the results obtained in our study indicate that assembled Wnt3a proteins, which interacted with each other in such a manner to indicate that the homo-trimer was the smallest unit, still possessed signaling activity. Again, the most critical difference between the previous studies and ours is whether or not the detergent was used for preparation of Wnt3a proteins. Thus, we speculate that these contradictory results can be explained if we suppose that Wnt proteins cannot maintain signaling activity and re-associate to form the trimer properly, once they are dissociated into the monomers by detergent.

Several studies have shown that Wnt proteins form complexes. Analysis by non-reducing gel electrophoresis showed that Wnt5a and Wnt11 form homodimers and heterodimers in *Xenopus* eggs, probably by forming disulfide bonds^32,33^. In contrast, mouse Wnt3a secreted from L cells rarely forms S–S-linked oligomers^30^. Consistently, the trimerization and assembly of Wnt3a shown in our study was not likely to have been due to intermolecular covalent bonding because the assembled Wnt3a was easily dissociated by interaction with the extracellular domain of Fzd8 or sFRP2. Of course, it is still possible that Wnt dimerization by disulfide linkage may be dependent on the Wnt subtype and/or cellular context; although further extensive analysis will be required to see whether native Wnt proteins are actually dimerized by disulfide linkage without performing any invasive procedure, including artificial concentration of protein samples

It is also known that N-terminally cleaved Wnt3a generated by a specific protease, Tiki, or C77A mutant form of Wnt3a, which is defective in an intramolecular S–S bridge, forms high-molecular-weight oligomers that are detectable by SDS-PAGE under non-reducing conditions^30^. In contrast, wild-type mouse Wnt3a predominantly migrated to the position corresponding to the monomer size in the presence of SDS even under non-reducing conditions, indicating that normal Wnt3a proteins are not covalently associated with each other^30^. In this study, we further showed that even wild-type Wnt3a form high-molecular-weight complexes, but that these complexes, could be easily dissociated by Fzd receptor or specific binding proteins such as sFRP2.

Although numerous studies reported various characteristics of Wnt proteins, most of these results were not derived under conditions where native characteristics of Wnt proteins were maintained. Therefore, the characteristics of Wnt proteins revealed in this present study give a closer reflection of those specific to native Wnt proteins. Nevertheless, we also note that the possibility cannot be excluded that the Wnt characteristics shown here may change depending on cellular context and Wnt subtype.

To understand the mechanism of Wnt-mediated tissue development, it is an intriguing question as to how Wnt proteins are transported among cells under the physiological condition. In this study, we showed the trimerization-mediated assembly to be an extracellular form of Wnt. Since this form of Wnt does not appear to include any serum component, it seems plausible that this form also exists in vivo. As shown by our experiments using *Xenopus* embryos, large Wnt3a assemblages were less mobile, suggesting that this form of Wnt would hardly be transported very far. However, the trimerization-mediated assembly probably makes it possible for dissociation of Wnt proteins, because the assembled forms of GFP-Wnt3a in the conditioned medium could be easily dissociated by the addition of Fzd8-CRD or sFRP2. As a result, the sFRP2-associated form of Wnt was more mobile and appeared to be transported across several cell diameters in *Xenopus* embryos. Therefore, we propose that conversion of Wnt complexes is a crucial event in the intercellular transport of Wnt. On the other hand, biochemical studies with cultured cells and tissues have shown that extracellular Wnt proteins associate with several binding proteins, sFRPs, SWIM and afamin, or membranous carriers, lipoprotein particles and exosomes^34^. One of the interesting questions is whether some of these binding proteins or membranous carriers can cause dissociation of the trimerization-mediated assembly and/or transport dissociated Wnt proteins. In addition, careful and extensive analyses is required to assess whether endogenous Wnt proteins are actually assembled in the trimerization-mediated manner, and if this is the case, it is also important to identify a Wnt transport system that is involved in the dissociation and transport of Wnt proteins in a particular tissue or cellular context of interest.

We recently showed that two different types of heparan sulfate proteoglycans (HSPG), which are separately clustered on the cell membrane, are differently associated with Wnt and sFRP^35^. While one type of the HSPG, which is enriched with N-sulfation on the heparin sulfate chain, is preferentially associated with Wnt8 and involved in the signaling, the other type, which is enriched with N-acetylation, is preferentially associated with Frzb, *Xenopus* sFRP3, and Wnt8/Frzb complexes. Thus, it seems plausible that Wnt3a trimers and Wnt3a/sFRP2 complexes may be differently associated with some particular types of HSPG on the cell membrane and that such interaction may cause different mobility of Wnt3a trimers and Wnt3a/sFRP2 complexes.

## Methods

### Cell culture and transfection

L and HEK293 cell lines, both of which were kindly provided by Dr. M. Takeichi, were maintained in Dulbecco’s modified Eagle’s medium (DMEM) or in a 1**:**1 mixture of DMEM and Ham’s F-12 medium supplemented with 8% fetal bovine serum (FBS) and antibiotics. L cells stably expressing GFP-tagged wild-type mouse Wnt3a (NM_009522), mutant Wnt3a (Wnt3a [C77A]), and *Drosophila* WntD (NM_142015.2) were established by the method indicated previously^24^. Plasmids for these Wnts were designed to express *Wnt* genes under the control of the PGK promoter, and a FLAG tag and monomeric EGFP (A206K)^36^ were fused to the end of the signal peptide sequence of each *Wnt* gene. For establishment of the secreting GFP-expressing cells, FLAG-tagged monomeric EGFP was fused to the end of the signal peptide of mouse Wnt3a under the control of the CMV promoter and was also introduced into L cells. Cells were selected and maintained in culture medium containing 400 μg mL^−1^ G418. L cells stably expressing wild-type Wnt3a and FLAG-tagged Wnt3a were established as previously described^1,24^. The supernatant from cultures of Wnt-producing cells was prepared as previously described^24^. *Fzd8* (NM_008058) *-CRD (1-235)-MycHis-* or *sFRP2* (NM_009144) *-FLAG-*expressing HEK293 cells under the control of the CMV promoter were also established. Cells were selected and maintained in culture medium containing 4 μg mL^−1^ Blasticidin S.

### Antibodies and western blotting

Monoclonal anti-Wnt-3a was described previously^1^. Western blotting was performed according to a standard protocol (see section “SDS-PAGE and Blue Native PAGE” below). Primary antibodies used for western blotting were as follows: Mouse monoclonal anti-Wnt-3a (1:2-1:20 dilution of hybridoma supernatant), mouse monoclonal anti-FLAG (M2-Alkaline phosphatase conjugated, SigmaA-9469, 1μg mL^-1^), and mouse monoclonal anti-β-catenin (5H10, Zymed B-8400, 0.5μg mL^-1^) antibodies. Secondary antibody used was anti-mouse IgG (HRP conjugated, Promega W402B, 0.25μg mL^-1^).

### Wnt3a activity assay

For assessment of Wnt activity by determining the β-catenin protein level, L cells (1.2 × 10^5^ cells) were introduced into 24-well culture plates 1 day before the activity assay. After removal of the culture medium, conditioned medium or 30 μL of each fraction separated by Superdex200 gel filtration chromatography was dissolved in a final volume of 600 μL medium and was added to the cells, which were then further incubated for 16 h. The cells were lysed in 40 μL of SDS-PAGE sample buffer, after which 8 μL of each lysate was subjected to 8% polyacrylamide gel for western blotting to detect β-catenin protein, an indicator of Wnt3a activity. STF293 cells (3 × 10^5^ cells), which stably express SuperTOPFlash reporter, were inoculated 1 day before treatment and used for the SuperTOPFlash assay. After removal of the culture medium, conditioned medium of GFP-Wnt3a/L cells prepared with FBS which has pre-immuno-depleted with anti-afamin or control antibodies was added to the cells, which were then further incubated for 6 h. Then, cells were harvested and monitored for luciferase activity with a luminometer. To examine specific activity (Wnt activity per net fluorescence (total fluorescence – background) shown in Fig. 1i), we measured the intensity of GFP fluorescence by using a fluorometer (Quantus, Promega). Because the amount of SuperTOPFlash vector was consistent between experiments, we did not conduct any additional normalization.

### Analytical ultracentrifugation

Media conditioned by GFP-tagged Wnt-expressing cells in FluoroBrite DMEM (Gibco) with 8%FBS were subjected to analytical ultracentrifugation (AUC; Beckman Coulter) with a fluorescence detection system (FDS; AVIV Biomedical). All AUC experiments were conducted by using Beckman 12-mm charcoal-filled Epon double-sector centerpieces at 20 °C and at a rotor speed of 42,000 r.p.m. Acquired data were analyzed by using a *c*(*s*) model of SEDFIT^37^. The resulting sedimentation coefficient distributions were transformed to standard conditions of water at 20 °C by considering the density, which was measured with an Anton Paar density meter DMA4500, and the viscosity of FluoroBrite DMEM with 8%FBS, which was measured with a Lovis 2000ME viscometer, respectively.

### Chromatography

Five-hundred mL of conditioned medium (C.M.) of FLAG-tagged Wnt3a (FLAG-Wnt3a)-expressing L cells in serum-free condition were prepared. HEPES buffer, pH7.4 (final 10 mM); EDTA, pH8.0 (final 1 mM); and protease inhibitors (final 1 mM PMSF, 5 μg mL^−1^ leupeptin, 10 μg mL^−1 ^ peptstatinA, and 5 μg mL^−1^ aprotinin) were added to the C.M. Debris was removed by centrifugation for 30 min at 14,000 × *g*, and the supernatant containing FLAG-Wnt3a was applied to an anti-FLAG M2 affinity gel (Sigma) column that had been equilibrated with 15 mM HEPES buffer, pH7.4-150 mM NaCl. The column was washed with 10 column bed volumes of the same buffer containing 0.1 mM n-Dodecyl-β-D-maltoside. The bound proteins were eluted with the same buffer containing 125 μg mL^−1^ FLAG peptide (Sigma). The eluate was concentrated 10–30 times with a Microcon centrifuge filter unit YM-100 (Millipore). Further purification was performed by using Superdex200 (PC3.2/30) gel filtration chromatography in a Smart-system (GE Healthcare). The elution of protein was profiled at 280 nm, and the eluate was sub-fractionated into 40-μL fractions.

### SDS-PAGE and Blue Native PAGE

SDS-PAGE was carried out by using the standard method of Laemmli^38^. Samples were mixed with a SDS-PAGE sample buffer and then heated at 60 °C for 5 min. Polyacrylamide gradient gel (SuperSep Ace, 10-20%; Wako Pure Chemicals) was used for electrophoresis. Proteins were visualized by silver staining (Wako) or Western blotting. For Western blots, proteins were transferred to a polyvinylidene difluoride (PVDF) membrane (Millipore) and detected with alkaline phosphatase-labeled anti-FLAG M2 antibody (Sigma).

Blue Native PAGE was carried out by using Native PAGE 3-12% Bis-Tris Gel (Invitrogen) in accordance with the manual of the manufacturer. After Blue Native PAGE, proteins were transferred to a PVDF membrane by using NuPAGE Transfer buffer (Invitrogen), fixed with 8% acetic acid for 15 min, and detected with anti-Wnt3a antibody and horseradish peroxidase-conjugated anti-mouse IgG (Promega), with Chemi-Lumi One Super (Nacalai tesque) used as a chemiluminescent substrate.

### Chemical cross-linking

For chemical cross-linking of Wnt3a, disuccinimidyl suberate (DSS) was added to a final concentration of 6.25, 12.5, 25, 50, or 100 μM; and the samples were incubated at 25 °C for 30 min. The cross-linking reaction was terminated by the addition of the SDS-PAGE sample buffer. Samples were incubated at 70 °C for 10 min, and then analyzed on NuPAGE 3–8% Tris-Acetate Gel (Invitrogen). After transfer to a PVDF membrane by using NuPAGE Transfer buffer, Wnt3a protein was detected with anti-Wnt3a antibody by the same method as used for Blue Native PAGE.

### Transmission electron microscopy

Wnt3a protein in fraction #14 separated by Superdex200 gel filtration chromatography was adsorbed by a thin carbon film supported by a copper mesh grid, negatively stained, and observed by using a JEOL 100CX transmission electron microscope at a magnification of 52,100 and 100 kV acceleration voltage. Images were recorded on SO-163 image films (Eastman Kodak, Rochester, NY), developed with a D19 developer (Eastman Kodak), and digitized with a Scitex Leafscan 45 scanner (Leaf systems Inc., Westborough, MA) at a pixel size of 1.92 Å at the specimen level. Wnt3a particle in fraction #14 was analyzed by electron tomography. The tomography of negatively stained Wnt3a protein was performed by using a scanning transmission electron microscopy (STEM) JEM2100F with a STEM-DFI detector (JEOL) at an acceleration voltage of 200-kV as previously described^39^.

### Automated particle selection and image analysis

Image analysis was performed with our SPINNS program^40^ and IMAGIC V^41^. Initially, 200 Wnt3a particles were selected and used to train a neural network (NN). With the trained NN, 4999 particles were selected, aligned, and sorted. Their class averages were adopted as new references, and this cycle was repeated. Euler angles of the class averages were determined by the echo-correlated three-dimensional reconstruction method with simulated annealing. Accordingly, we hypothesized that the symmetry of the major Wnt3a particles was either C3 or D3. Therefore, 2 independent analyses of the same particle library were employed using both the C3 and D3 symmetries. These were separately imposed in the subsequent computations. A primary three-dimensional density map was calculated and refined, also in cycles. We chose the C3 symmetric volume as a final structure based on higher correlation of reprojections with the averaged images, and with the original raw images. To assess the resolution of the stable 3D density map, we divided the data into odd and even subsets. Using these subsets, 2 independent 3D reconstructions were computed without masking. These two 3D density maps were compared by Fourier shell correlation at the threshold of 0.5, using IMAGIC V^41^.

### Experiments with *Xenopus* embryos

All experiments using *Xenopus laevis* were approved by the Institutional Animal Care and Use Committee, National Institutes of Natural Sciences. This study was performed in accordance with the guidelines for Animal Experimentation of National Institutes of Natural Sciences, and all efforts were made to minimize suffering during experimental procedures. Artificial fertilization, manipulation of *Xenopus*, and microinjection of *Xenopus* embryos were carried out as described^9,42^. mRNAs were microinjected into the animal pole region of ventral blastomeres of 4-cell stage embryos. Amounts of injected mRNAs are indicated as follows: For FCCS experiments, 1000 pg of *mCherry* (AY678264)-*Wnt3a* mRNA was injected into an embryo with 500 pg of mRNA of *GFP-Wnt3a* or other GFP-constructs. In addition, 1000 pg of *Fzd8-CRD* or *sFRP2* mRNA was also injected with 1000 pg of *mCherry-* and 500 pg of *GFP-Wnt3a* mRNAs in the experiments shown in Fig. 5a. For FCS and immunostaining experiments, 250 pg of *GFP-Wnt3a* mRNA and 1000 pg of *sFRP2* mRNA were injected into an embryo. Immunostaining and *in situ* hybridization were carried out as described^9,35^. Gal staining was carried out with Magenta-Gal (5-bromo-6-chloro-3-indolyl b-D-galactopyranoside, Carbosynth, UK).

### FCS and FCCS analyses

Fluorescence Correlation Spectroscopy (FCS) and Fluorescence Cross-Correlation Spectroscopy (FCCS) analyses were carried out on *Xenopus* embryos at the gastrula stage (stage 10.5–11.5). Embryos were mounted on glass-based dishes (IWAKI) with 1.5% low melting point agarose (Invitrogen) in 0.1x Steinberg’s solution without phenol red. We performed FCS and FCCS at boundaries between non-Wnt3a-expressing cells within almost one-cell diameter from Wnt3a-expressing cells, especially aiming at the basolateral intercellular space. We found no signal of molecules diffusing from the apical extracellular space. We confirmed that only very slow autocorrelation of fluorescent signal was detected within Wnt-expressing and -receiving cells. Since this slow correlation was apparently different from the fast autocorrelation of diffusion, we could also distinguish our measurement in the extracellular space from that within the cell by the pattern of autocorrelation curves. On the other hand, distinction of molecular movements between the extracellular space and on the cell membrane is intrinsically difficult. However, we only chose measurements that detected autocorrelation curves corresponding to fast molecular diffusion, which ensures that the measured volume contains the extracellular space.

FCS and FCCS measurements were performed by using a confocal microscope system (LSM510; Carl Zeiss) combined with a ConfoCor2 (Carl Zeiss). Details of the combined microscopic system and analysis of fluorescence auto- and cross-correlation functions obtained from FCS and FCCS measurement were described in previous studies^43–45^. Briefly, the fluorescence correlation functions (FCF, *G*(τ)), from which absolute number and diffusion coefficient (*D*) of mobile fluorescent molecules, fractional ratio, and interaction amplitude represented by relative cross-correlation amplitude (RCA) were calculated, were obtained as follows:

The fluorescence auto-correlation functions of the red and green channels, *G*_r_(*τ*) and *G*_g_(*τ*), and the fluorescence cross-correlation function, *G*_c_(*τ*), were calculated from

1 $$G_x\left( \tau \right) = 1 + \frac{\langle{\delta I_i(t) \cdot \delta I_j(t + \tau )}\rangle}{{\langle{I_i\left( t \right)} \rangle\langle {I_j\left( t \right)} \rangle}},$$

where *τ* denotes the time delay; *I*_*i*_ the fluorescence intensity of the red channel (*i* *=* r) or green channel (*i* = g); and *G*_r_(*τ*), *G*_g_(*τ*), and *G*_c_(*τ*) denote the red (*i* *=* *j* *=* *x* *=* r) and green (*i* *=* *j* *=* *x* *=* g) auto-correlation functions and the cross-correlation function (*i* *=* r*, j* *=* g, *x* *=* c), respectively. The acquired *G*_*x*_(*τ*) values were fitted by using a one-component model for solution sample and a two- or three-component model for *Xenopus* embryos:

2 $$G_x\left( \tau \right) = 1 + \frac{1}{N}{\sum }_{i} F_i\left( {1 + \frac{\tau }{{\tau _i}}} \right)^{ - 1}\left( {1 + \frac{\tau }{{s^2\tau _1}}} \right)^{ - 1/2},$$

where *F*_*i*_ and *τ*_*i*_ are the fraction and diffusion time of component *I*, respectively; *N* is the average number of fluorescent particles in the excitation-detection volume defined by the radius *w*_0_ and the length 2*z*_0_; and *s* is the structure parameter representing the ratio *s* *=* *z*_0_*/w*_0_.

To estimate the diffusion coefficient and fractional ratio from FCS measurements of GFP-Wnt3a, FCFs in *Xenopus* embryos were fitted by a two-component model (*i*=2, *D*_fast_ and *D*_slow_) with a triplet term.^43^ Averaged fluorescence intensity per molecule (count per molecule, CPM) was calculated from averaged fluorescence intensity and number of molecules. For FCCS measurements, simultaneous excitation of GFP- and mCherry-tagged Wnt3a proteins was carefully carried out at minimal and optimal excitation powers, chosen to obtain sufficiently high signal-to-noise ratios for analysis of the diffusional coefficient and molecular interaction. Data containing severe photobleaching possibly resulting from a high proportion of immobilized fluorophores and non-stationary fluorescent signals resulting from drift of embryos were excluded from the analysis. All measured FCFs in solution samples and cells were fitted by analytical software installed on the microscopic system, using the model described above.^43–45^

### Quantification and statistical analysis

Quantification and statistical tests employed for each experiment are indicated in the figure legends. For all data in these analyses, *p* values of less than 0.05 were considered to be significant. Statistical analyses were performed using R software (version 3.4.1). GUSSI software^46^ was employed for quantification of peak areas detected by AUC-FDS analyses.

## Electronic supplementary material

Supplementary file

## Data Availability

Structural data of Wnt3a complex have been deposited in the Electron Microscopy Data Bank (www.emdatabank.org) under the following accession numbers: EMD-9628. The authors declare that all data supporting the findings of this study are available within the article and its Supplementary Information files or from the corresponding author upon reasonable request.
