# Supplementary material for: Assembly of protein complexes restricts diffusion of Wnt3a proteins
Source: Commun Biol. 2018 Oct 10;1:165. doi: 10.1038/s42003-018-0172-x (PMC6179999; doi:10.1038/s42003-018-0172-x)
Supplement: Supplementary file 1 — Supplementary file [file 42003_2018_172_MOESM1_ESM.pdf]

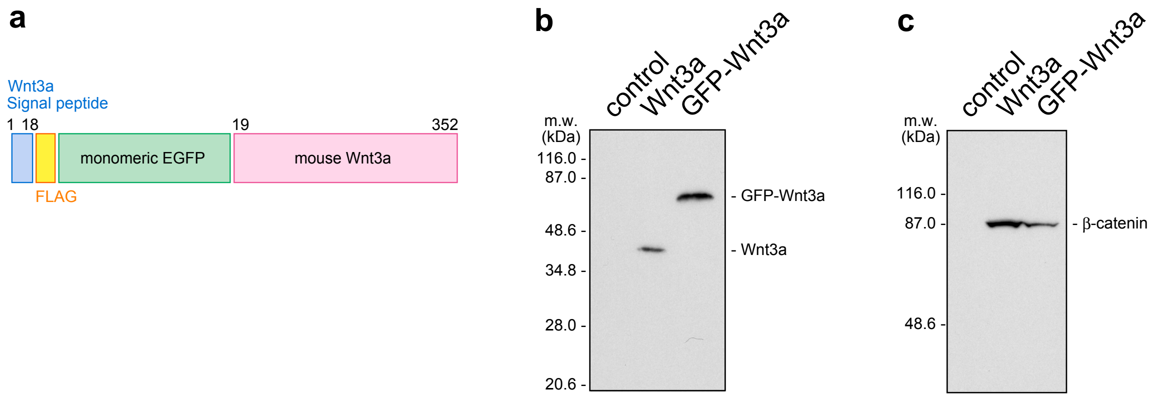

Supplementary Figure 1. Activity of GFP-tagged Wnt3a.
a, Schematic diagram of GFP-Wnt3a protein. **b**, The amount of secreted Wnt3a was examined by Western blotting. **c**, Signaling activity of GFP-Wnt3a secreted in the conditioned medium was measured by stability of β-catenin in L cells.

a

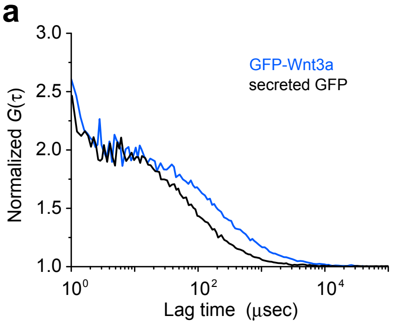

b

	GFP-Wnt3a	secreted GFP
Diffusion time (µsec)	234 ± 26	83 ± 11
D value ($\mu m^2\ sec^{-1}$)	25 ± 3	72 ± 10
Estimated m.w. (kDa)	~ 400	28

c

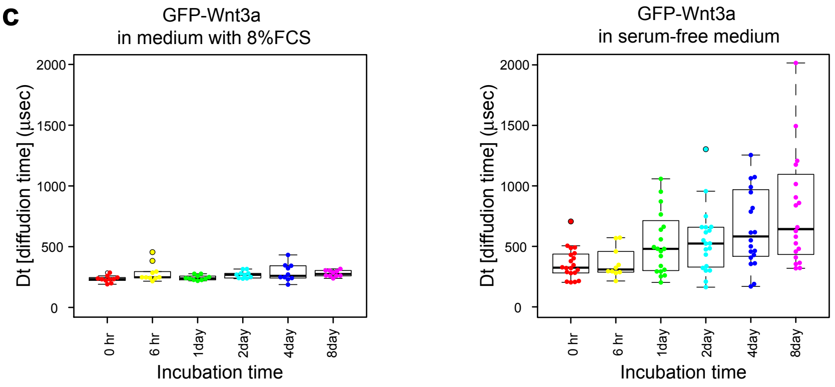

Supplementary Figure 2. Fluorescence Correlation Spectroscopy (FCS) analysis to assess the sizes of GFP-Wnt3a in the conditioned medium.

a, Normalized fluorescence correlation function (FCF) curves for GFP-Wnt3a (blue) and a secreted form of monomeric GFP (secreted GFP, black) in conditioned medium. **b**, The sizes of GFP-Wnt3a proteins in conditioned medium were estimated by FCS analysis. The average size of GFP-Wnt3a in the conditioned medium was determined by comparing the diffusion coefficient of fluorescent particles with that of secreted GFP. The apparent average size of GFP-Wnt3a was estimated to be about 400 kDa, which was larger than the predicted size for the monomeric GFP-Wnt3a (70 kDa). **c**, Change in average sizes of GFP-Wnt3a during incubation in conditioned medium with or without serum. Average sizes of GFP-Wnt3a in serum-free media, which are proportional to its diffusion time (Dt), gradually increased during the incubation at 37˚C for 8 days, whereas those in conditioned medium containing serum was not obviously changed during the incubation.

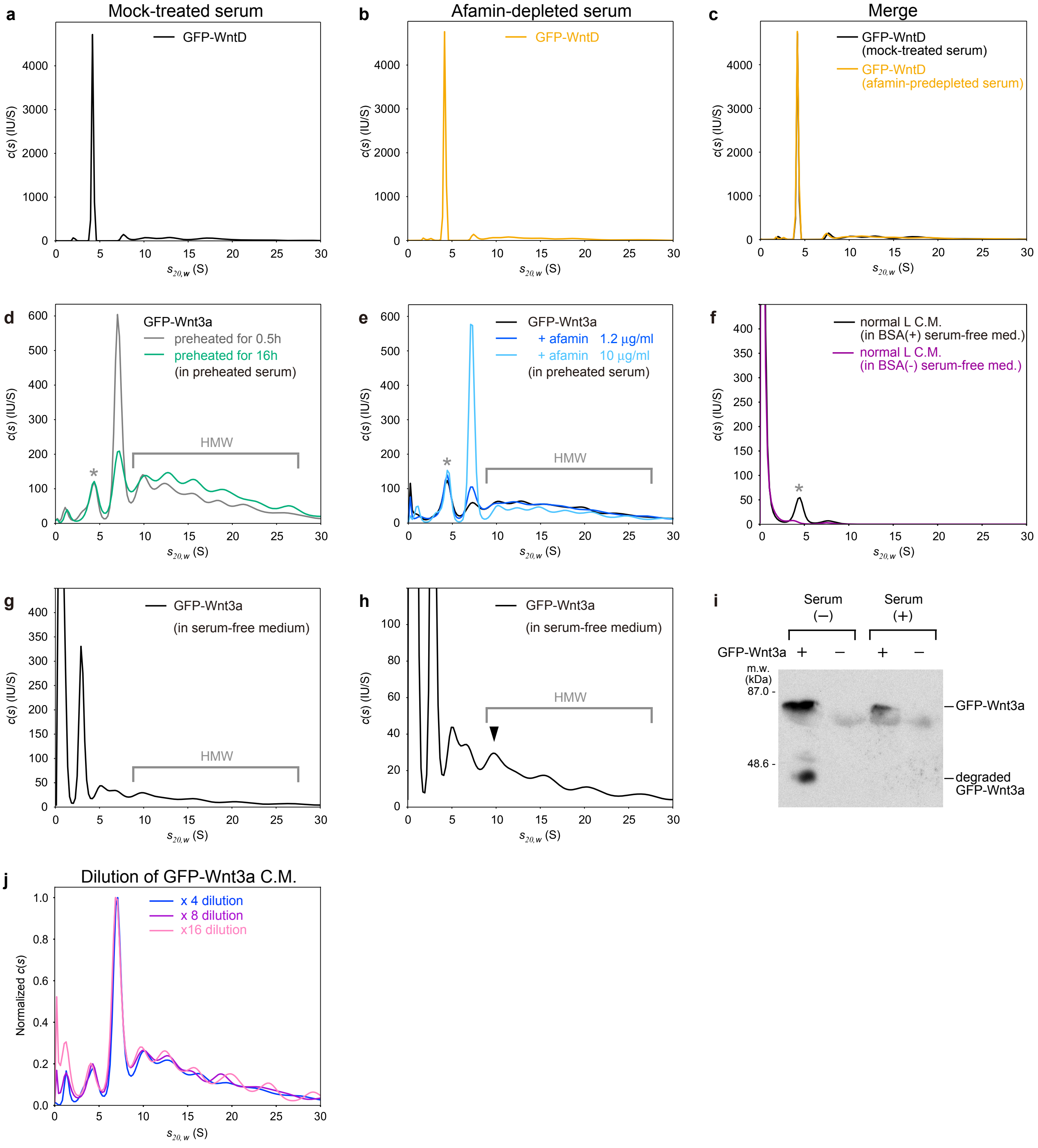

Supplementary Figure 3. Effect of serum protein on the analytical ultracentrifugation analyses of GFP-tagged Wnt3a proteins.

a-c, Effect of afamin-depletion on the result of analytical ultracentrifugation analysis of GFP-tagged WntD proteins secreted from cultured cells. AUC-FDS analysis of GFP-WntD in culture media with mock-treated (**a**) and afamin pre-depleted (**b**) serum. Merged figures are also indicated (**c**). **d**, AUC-FDS analysis of GFP-Wnt3a in the culture media in which the serum was pre-inactivated by heat treatment (n=6). The area under the 7.0 S peak was diminished by preincubation of serum at 56˚C prior to cultivation with GFP-Wnt3a-producing cells. **e**, AUC-FDS analysis of GFP-Wnt3a in the culture media in which human afamin was added back to heat-inactivated serum (n=2). As previously reported[1], the addition of afamin increased the amount of Wnt proteins released in the culture medium. Most of these Wnt proteins increased in amount formed complexes with afamin. **f**, AUC-FDS analysis of the media conditioned by normal L cells cultured in serum-free media with no (purple line) or 1mg mL^{-1} (black line) of BSA for 2 days after serum removal (n=2). **g**, **h** , AUC-FDS analysis of GFP-Wnt3a in the serum-free media recovered 2 days after serum removal (n=6). **h** is an enlarged figure of **g**. HMW indicates the high-molecular-weight complex. The closed arrowhead in **h** indicates the smallest HMW complex. Peaks marked with asterisks in **d** , **e** , and **f** appeared to be derived from the fluorescent molecules bound to serum albumin, such as bilirubin. Note that this peak was not apparent in **g**. **i**, Western blotting results for GFP-Wnt3a proteins released into the conditioned media in the absence or presence of serum. Some GFP-Wnt3a molecules were degraded in the serum-free condition. **j**, Effect of dilution of the conditioned medium of GFP-Wnt3a on the results of the AUC-FDS analysis. Conditioned medium of GFP-Wnt3a/L cells was diluted with serum-free medium and subjected to AUC-FDS analysis. Since the peak position of the major peak at ~7.0S did not change by dilution of the conditioned media, the concentration of Wnt proteins did not affect the assignment of the molecular weight of this peak. In contrast, the positions of the peaks of the HMW complex were shifted as the conditioned media were diluted. Since only the peak position of the smallest molecular mass was not significantly changed among these peaks, we estimated only the moleclar weight of this peak.

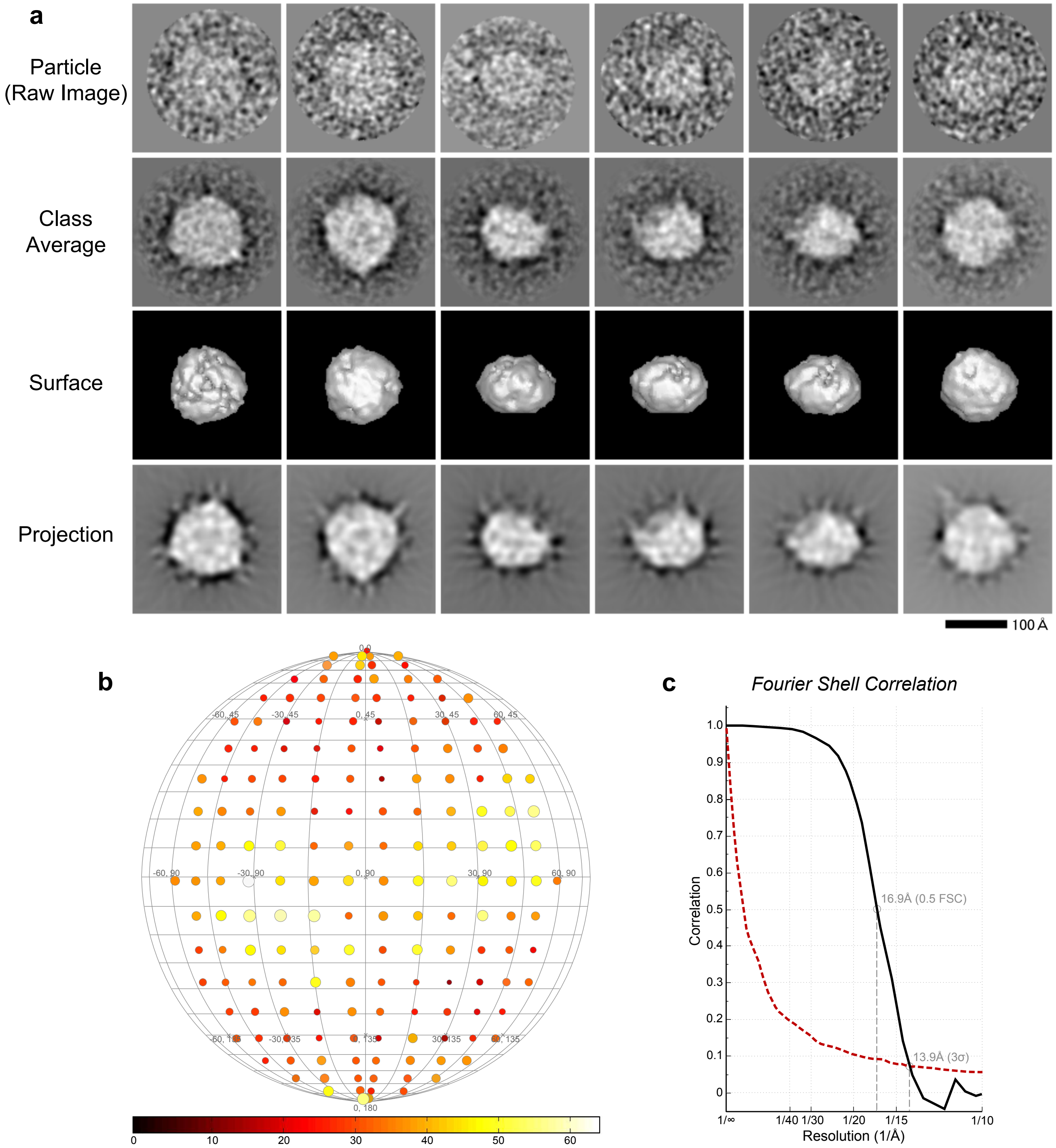

Supplementary Figure 4. Procedure for single-particle analysis.

a, Raw particle images of Wnt3a in fraction 14 with different Euler angles (top row) are compared with the corresponding two-dimensional class averages (second row), the surface views of the three-dimensional reconstruction (third row), and the reprojections of the three-dimensional reconstruction (fourth row) along the corresponding Euler directions. They are consistent through the reconstruction. The scale bar represents 100 Å. **b**, The Euler angle (β, γ) distribution of class average images. The distribution covers the whole angular range. **c**, Fourier shell correlation function indicates a resolution limit of 16.9 Å by the FSC>0.5 criterion.

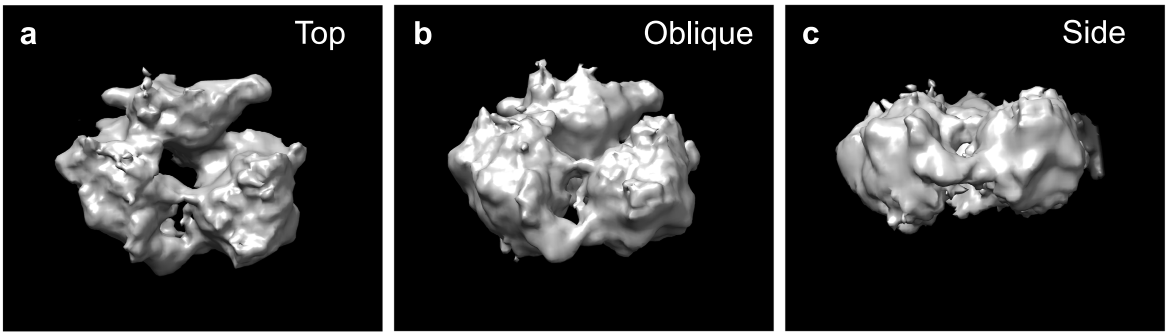

Supplementary Figure 5. Image of Wnt3a reconstructed by electron tomography.
One typical particle recovered in fraction 14 was analyzed by electron tomography. The tomography of negatively stained FLAG-Wnt3a was performed by using a scanning transmission electron microscopy (STEM) with a STEM-DFI detector. Top view (**a**), oblique view (**b**), and side view (**c**) are shown. This reconstructed image shows a three-fold rotational symmetrical structure.

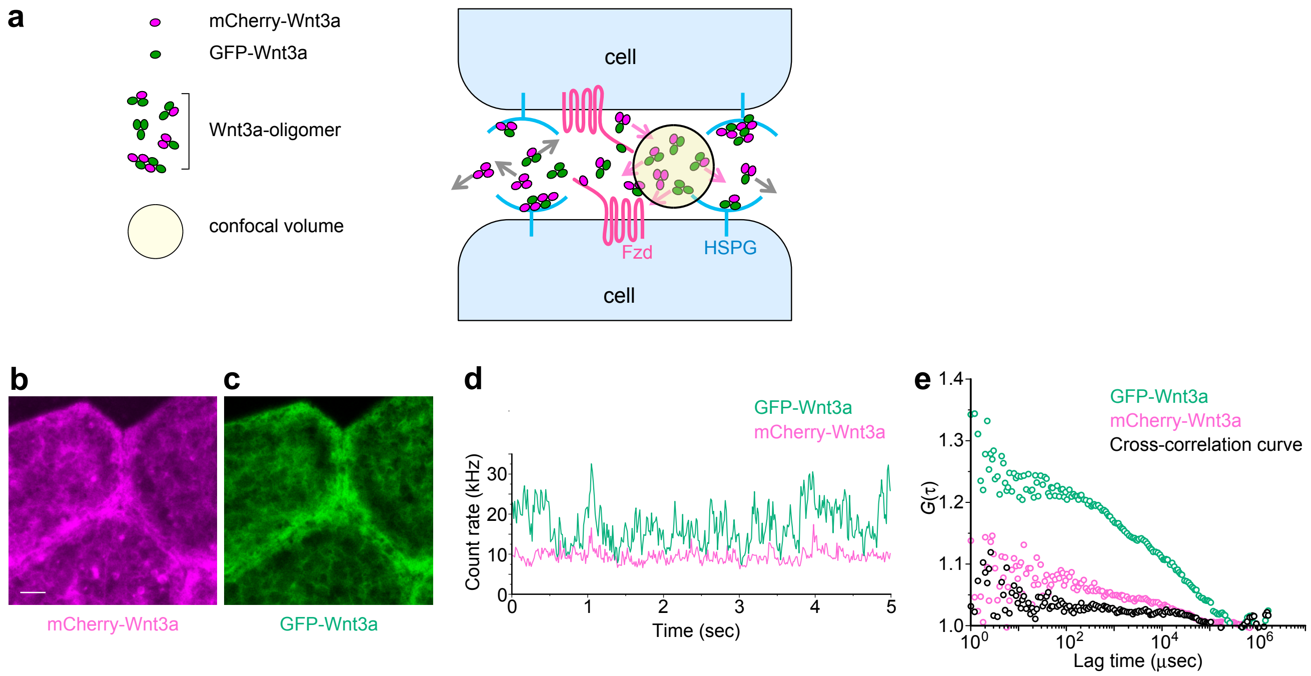

Supplementary Figure 6. Fluorescence Cross-Correlation Spectroscopy (FCCS) analysis of mCherry-Wnt3a and GFP-Wnt3a in the extracellular milieu in *Xenopus* embryos.

a, Schematic representation of the FCCS analysis is presented. **b**, **c**, Fluorescent images of mCherry-Wnt3a (**b**) and GFP-Wnt3a (**c**) in *Xenopus* are shown. **d**, An example of raw fluorescence fluctuation data for the extracellular milieu of a *Xenopus* embryo which was injected with both *mCherry-Wnt3a* (magenta) and *GFP-Wnt3a* (green) mRNAs in the same blastomere is shown. **e**, Auto-correlation curve of GFP-Wnt3a (green) and mCherry-Wnt3a (magenta), and calculated cross-correlation curve (black) between GFP-Wnt3a and mCherry-Wnt3a are also shown. The scale bar in **b** represents 5 μm.

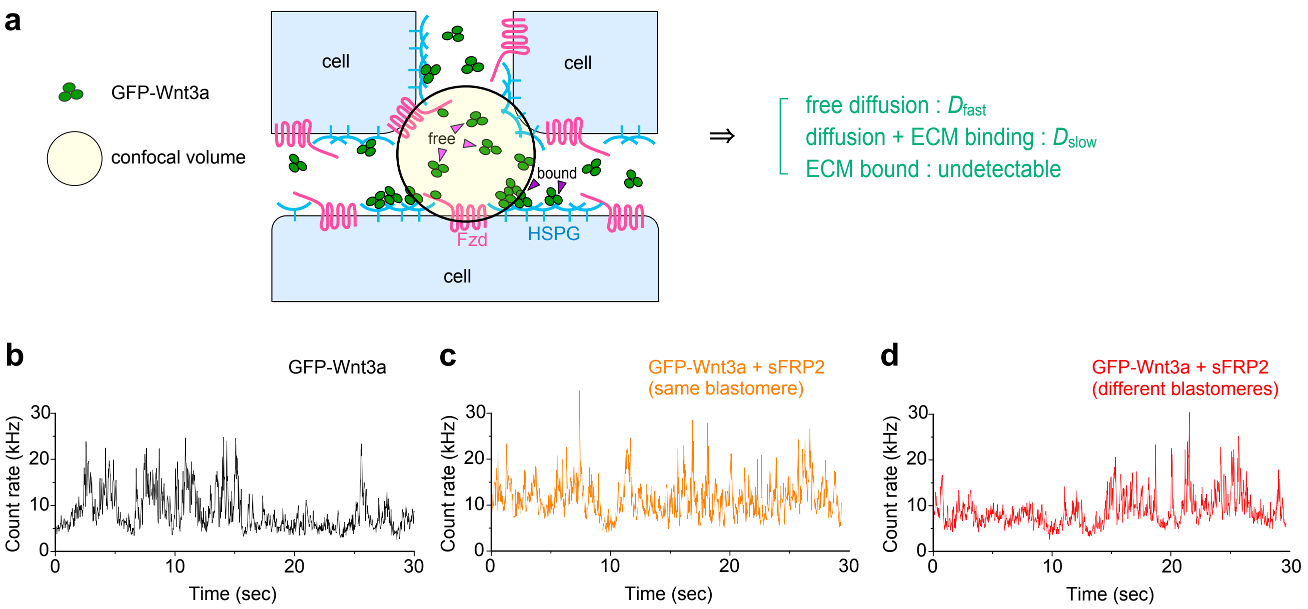

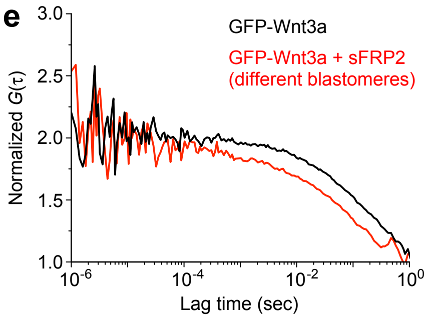

f

		GFP-Wnt3a	GFP-Wnt3a+sFRP2 (same blastomere)	GFP-Wnt3a+sFRP2 (different blastomeres)
Slow component	Fractional ratio (%)	81	76	83
	D_{slow} (μm^2 sec^{-1})	0.09 ± 0.08	0.12 ± 0.10	0.11 ± 0.10
Fast component	Fractional ratio (%)	19	24	17
	D_{fast} (μm^2 sec^{-1})	12.6 ± 8.8	16.2 ± 22.8	14.4 ± 10.2
Number of particle		4.3 ± 3.3	10.0 ± 4.5	7.2 ± 5.2
Number of measurement		127	118	136

Supplementary Figure 7. Fluorescence Correlation Spectroscopy (FCS) analysis of GFP-Wnt3a with sFRP2 in the extracellular milieu in *Xenopus* embryos.

a, Schematic representation of the FCS analysis is presented. **b-d**, Examples of raw fluorescence fluctuation data obtained from *Xenopus* embryos injected with *GFP-Wnt3a* mRNA only (**b**), with *GFP-Wnt3a* and *sFRP2* mRNAs into the same blastomere (**c**) or injected with *GFP-Wnt3a* and *sFRP2* mRNAs into different blastomeres (**d**) at the 4-cell stage and then analyzed by FCS at mid-gastrula stage are shown. **e**, Normalized FCF curves of GFP-Wnt3a (black) and GFP-Wnt3a with sFRP2 (red, in the case of injection into different blastomeres as shown in **d**) are also shown. **f**, Diffusion coefficient and number of particles (equivalent to the concentration) were also calculated and shown in the table.

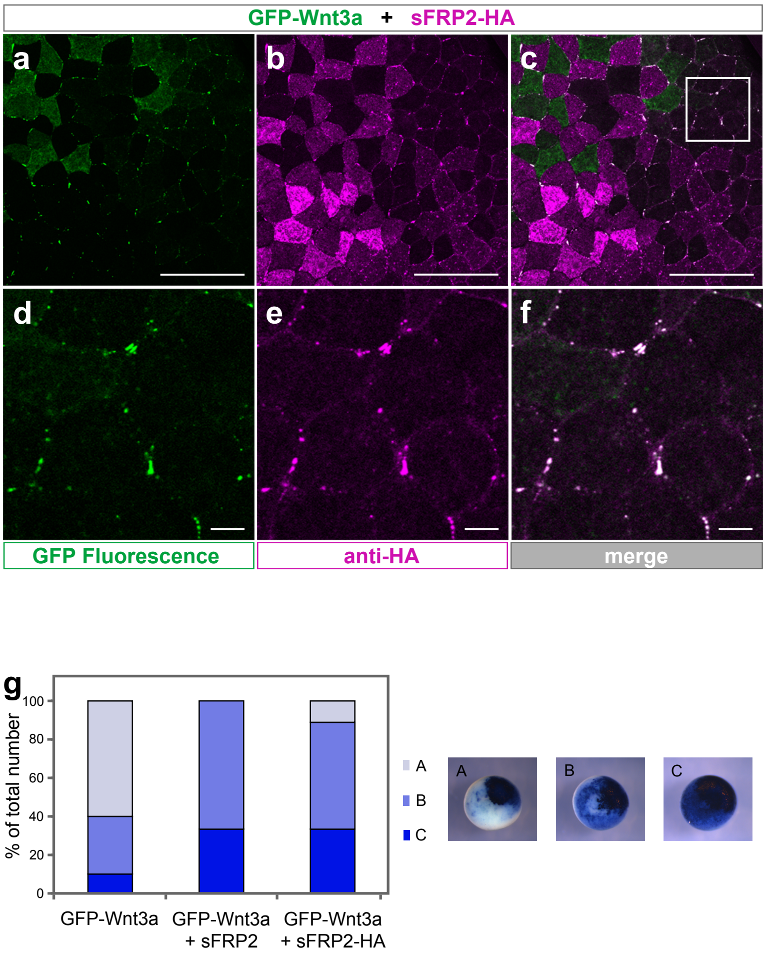

Supplementary Figure 8. Expansion of Wnt3a distribution range by expression of sFRP2 in *Xenopus* embryos.

a-f, Distribution of GFP-Wnt3a and sFRP2 in *Xenopus* embryo, observed by GFP-fluorescence (**a**, **d**) and immunostaining with anti-HA and Alexa555-labeled secondary antibody (**b**, **e**). *GFP-Wnt3a* and *sFRP2-HA* mRNAs were injected into different blastomeres in *Xenopus* embryos at the 4-cell stage, and the distribution of both proteins were observed at stage 10.5 (n=4). When sFRP2 was present, Wnt3a and sFRP2 were clearly co-localized (**c**, **f**). **d-f** are magnified photos of the area surrounded by the white square in **c** . The scale bars in **a-c** represent 100 µm; and those in **d-f** , 10 µm. **g**, Immunostaining of GFP-Wnt3a with sFRP2 in *Xenopus* embryos. Distribution of GFP-Wnt3a in the absence (n=10), presence of sFRP2 (n=9), or sFRP2-HA (n=9) expression. GFP-Wnt3a was visualized by using anti-GFP and alkaline phosphatase-conjugated secondary antibody. The stained embryos were classified into 3 levels, showed in “A” to “C” . The number of embryos for each level was counted and summarized. The range of the Wnt3a-distribution was expanded by expression of sFRP2 in *Xenopus* embryos.

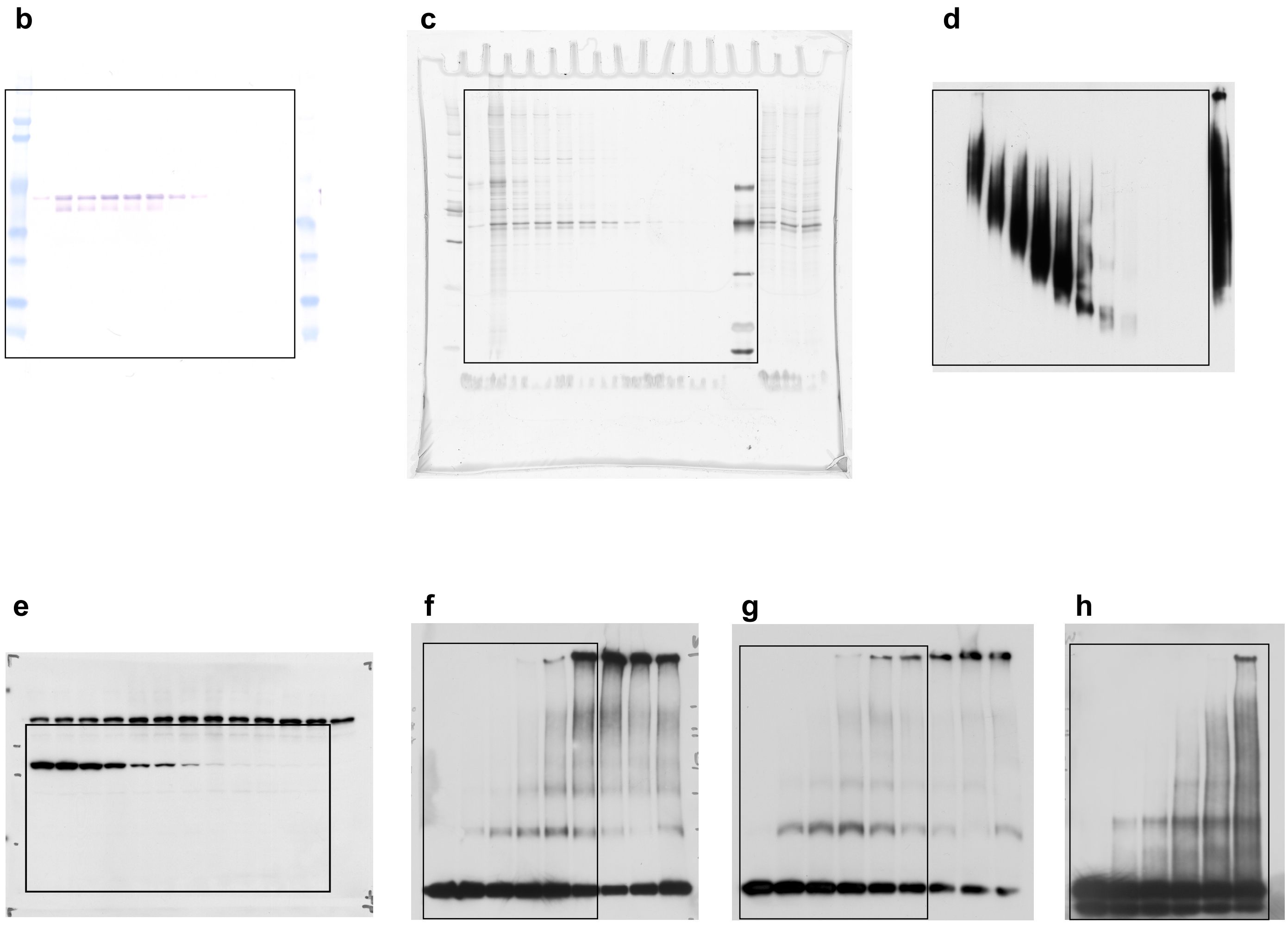

Supplementary Figure 9. Full blot images for Figure 3 b-h.

Supplementary References

1 Mihara, E. et al. Active and water-soluble form of lipidated Wnt protein is maintained by a serum glycoprotein afamin/α-albumin. *Elife* **5**, doi:10.7554/eLife.11621 (2016).
